# De novo creation of a naked eye–detectable fluorescent molecule based on quantum chemical computation and machine learning

**DOI:** 10.1126/sciadv.abj3906

**Published:** 2022-03-09

**Authors:** Masato Sumita, Kei Terayama, Naoya Suzuki, Shinsuke Ishihara, Ryo Tamura, Mandeep K. Chahal, Daniel T. Payne, Kazuki Yoshizoe, Koji Tsuda

**Affiliations:** 1 RIKEN Center for Advanced Intelligence Project, 1-4-1 Nihonbashi, Chuo-ku, Tokyo 103-0027, Japan.; 2 International Center for Materials Nanoarchitectonics (WPI-MANA), National Institute for Materials Science, 1-1 Namiki, Tsukuba, Ibaraki 305-0044, Japan.; 3 Graduate School of Medical Life Science, Yokohama City University, 1-7-29, Suehiro-cho, Tsurumi-ku, Kanagawa 230-0045, Japan.; 4 Graduate School of Medicine, Kyoto University, 53 Shogoin-Kawaharacho, Sakyo-ku, Kyoto 606-8507, Japan.; 5 Medical Sciences Innovation Hub Program, RIKEN Cluster for Science, Technology and Innovation Hub, Tsurumi-ku, Kanagawa 230-0045, Japan.; 6 Materials Science and Engineering, Osaka Prefecture University, 1-1 Gakuen-cho, Nakaku, Sakai, Osaka 599-8531, Japan.; 7 Research and Services Division of Materials Data and Integrated System, National Institute for Materials Science, 1-1 Namiki, Tsukuba, Ibaraki 305-0044, Japan.; 8 Graduate School of Frontier Sciences, The University of Tokyo, 5-1-5 Kashiwa-no-ha, Kashiwa, Chiba 277-8561, Japan.; 9 Department of Chemistry, University of Southampton, University Road, Highfield, Southampton SO17 1BJ, UK.; 10 International Center for Young Scientists (ICYS), National Institute for Materials Science, 1-1 Namiki, Tsukuba, Ibaraki 305-0044, Japan.; 11 Research Institute for Information Technology (RIIT), Kyushu University, 744 Motooka, Nishi-ku, Fukuoka City, Fukuoka 819-0395, Japan.

## Abstract

Designing fluorescent molecules requires considering multiple interrelated molecular properties, as opposed to properties that straightforwardly correlated with molecular structure, such as light absorption of molecules. In this study, we have used a de novo molecule generator (DNMG) coupled with quantum chemical computation (QC) to develop fluorescent molecules, which are garnering significant attention in various disciplines. Using massive parallel computation (1024 cores, 5 days), the DNMG has produced 3643 candidate molecules. We have selected an unreported molecule and seven reported molecules and synthesized them. Photoluminescence spectrum measurements demonstrated that the DNMG can successfully design fluorescent molecules with 75% accuracy (*n* = 6/8) and create an unreported molecule that emits fluorescence detectable by the naked eye.

## Teaser

An AI system combined with quantum chemistry successfully designed several unreported fluorescent compounds.

## INTRODUCTION

Fluorescent compounds are important as visible photoemitters in applications across several disciplines, including organic light-emitting diodes (*1*–*4*), sensors (*5*–*8*), and bioimaging (*9*–*11*). Although numerous fluorescent molecules have been developed for these and other applications, new ones are in constant demand to address the shortcomings of current materials in terms of function, sustainability, and low cost. Even subtle changes in chemical structures might lead to major improvements. Fluorescence is a photochemical property governed by quantum mechanics. However, despite the long history of the study of fluorescence, there are no clear guidelines for creating the fluorescent molecules as there are for light-absorbing ones (*1*, *5*, *9*).

**Fig. 1.**
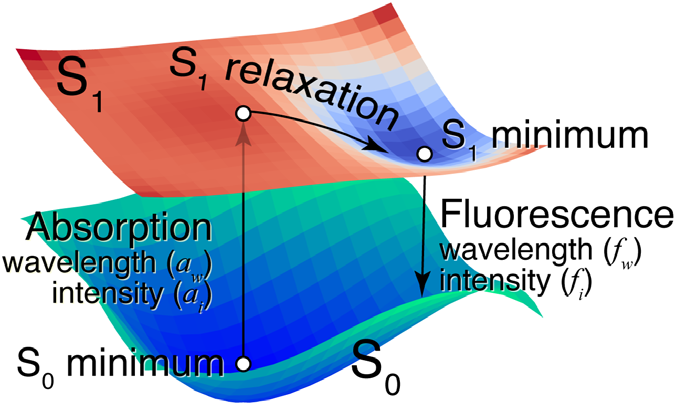
Schematic of the PESs of the singlet ground (S_0_) state and the singlet first excited (S_1_) state of a fluorescent molecule. At the S_0_ minimum, the molecule is excited vertically to the S_1_ excited state, absorbing light of wavelength *a_w_* with the intensity *a_i_*. Relaxing in the S_1_ state, the molecule reaches the S_1_ minimum and fluoresces with wavelength *f_w_* and intensity *f_i_*.

A simplified physicochemical mechanism of fluorescence emission from a molecule is illustrated in Fig. 1. Initially, we consider the molecule to be in its singlet ground (S_0_) state; at the S_0_ minimum, it absorbs light and transits to the first excited singlet (S_1_) state. The S_1_ excited molecule relaxes to a minimum in the S_1_ state and back to the S_0_ state, emitting the energy difference between the S_1_ and S_0_ states as light (fluorescence). The excited molecule should travel to the minimum in the S_1_ state for emitting light without deactivation. Several factors, including reactions with oxygen molecules, molecular collisions, intra/intermolecular electron transfer, and aggregation, may deactivate the molecule as it travels in the excited state (*12*). This makes it difficult to correlate fluorescence with molecular structure. Hence, automatizing fluorescent molecule design would be helpful.

Recently, de novo molecule generators (DNMGs) based on machine learning (ML) (*13*–*16*) have been developed for designing molecules with simple and predictable values such as the logarithm of the partition coefficient (log*P*), which can be estimated from the constitute parts of a molecule (*17*–*19*). Combining the DNMGs with classical simulations has successfully generated molecules with improved versatility and practicality. For instance, the combination of a DNMG and docking simulation can be used to design biologically active molecules; this was investigated by organic syntheses (*20*). In combination with molecular dynamics or predictive models, DNMGs can also direct the synthesis of functional polymers (*21*, *22*).

In previous study, we have combined quantum chemical computations (QCs) with our DNMG, called ChemTS (*23*), which (in principle) can design functional molecules characterized by their quantum mechanical (QM) properties de novo (*24*). Thus, ChemTS coupled with QC was applied to design molecules that can absorb light with a desired wavelength (*24*). Among the 86 designed and generated molecules, 6 known molecules that were not included in the training dataset were selected for the ultraviolet-visible (UV-vis) absorption measurement. The results were consistent with the target wavelength of the generator. In addition, DNMGs increase the possibility of discovering new molecules because the search area of a DNMG is not limited in the dataset in contrast to the traditional high-throughput QM and screening with ML models. We also performed functional group enrichment analysis of the molecules produced by ChemTS with QC to maximize the electron gain energy and found important functional groups that are not included in the electret literature (*25*).

Although relatively simple properties, such as light absorption and electron gain energy, can be straightforwardly correlated with molecular structure, complex phenomena such as fluorescence that are exhibited only by specific molecules pose vastly greater difficulty. In the case of fluorescence, as shown in Fig. 1, it is necessary to consider multiple properties, which are intricately intertwined. This makes it difficult to establish intuitive guidelines for the design of molecular structures. To design practically useful compounds, the complex mechanisms governing the target molecular property must be appropriately digitized for utilization in DNMGs. Moreover, the growth of computational cost with increasing the complexity of the mechanism to explore chemical space must be considered.

In this study, we designed fluorescent molecules with a massively parallelized version of ChemTS. The program package used QC to digitize the minimum requirements of the fluorescence mechanism (Fig. 1). There are several user-friendly software packages (*26*–*28*) based on electronic structure theories that can be used for QC on molecules and materials. Balancing reliability against computational costs, we used density functional theory (DFT) (*29*) to evaluate the potential energy surfaces (PESs) shown in Fig. 1. To solve the computational costs of extensively exploring the chemical space, we parallelized ChemTS massively to use 1024 cores based on the concept of virtual loss and generated 3643 molecules. For validation, we synthesized an unreported compound and several reported ones from those generated. Six compounds, including an unreported one, fluoresced as expected. The unreported molecule, which is synthesizable through coupling between commercially available reagents, has an unexpected property, although it is composed of common fragments [coumarin (*5*), pyridine (*30*), and pyrazolopyrimidine (*31*)]. This suggests that the massive parallel DNMG has the potential to induce a paradigm shift in the molecular design. Our implementations of the digitalized mechanism of fluorescence and the parallelized DNMG are available at https://github.com/tsudalab/GaussianRunPack and https://github.com/tsudalab/FL_ChemTS.

## RESULTS

### Molecule generation

**Fig. 2.**
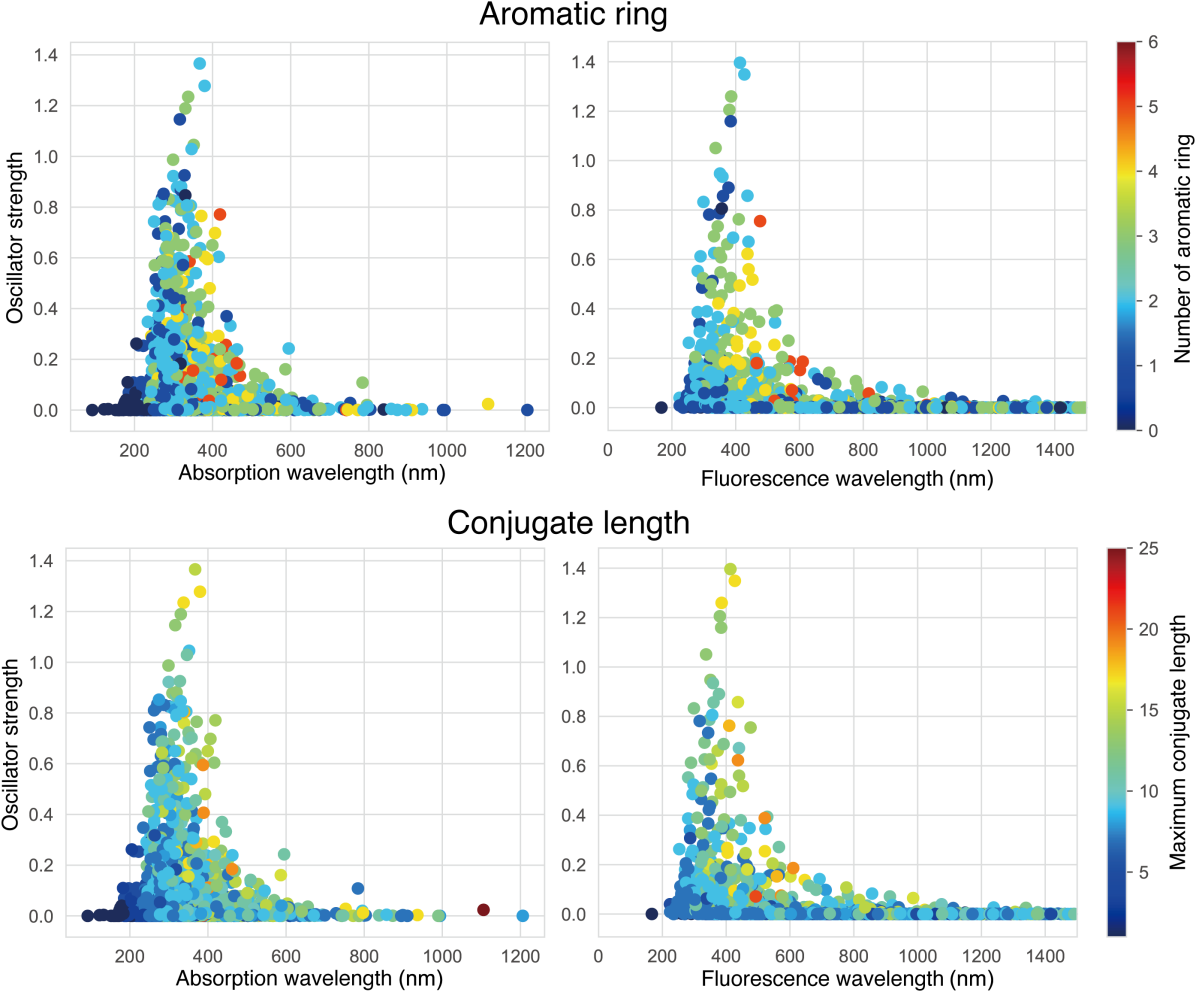
Distribution profiles of OSs for absorption to S_1_ states and fluorescence from S_1_ states of the generated molecules at the B3LYP/3-21G* level. In the (**top**) images, the colors indicate the number of aromatic rings; in the (**bottom**) images, they indicate the conjugate length.

Assuming that fluorescent molecules could be used for bioimaging (*10*), we set the upper limits of the absorption and fluorescence wavelengths at 700 and 1200 nm, respectively, and the lower limits of the oscillator strengths (OSs) at 0.01. ChemTS designed 3643 candidate fluorescent molecules using 1024 cores for 5 days. The distribution profiles of the absorption and fluorescence wavelengths and OSs of the molecules evaluated by DFT are shown in Fig. 2 with the structural characteristics, i.e., the number of aromatic rings and the conjugate length. Their theoretical S_1_ absorption wavelengths were distributed from 120 to 1200 nm; the fluorescence wavelengths from their S_1_-minimum states were all >180 nm. The molecules with high OS for adsorption and fluorescence were distributed from approximately 200 to 600 nm.

Typical chromophores often feature the conjugation of multibonds and aromatic rings in organic molecules (*1*). To determine whether this holds for the candidate molecules produced by the generator, we have analyzed the number of aromatic rings and the conjugate lengths. In Fig. 2, the colored points representing various numbers of aromatic rings are scattered over the entire distribution. However, the brightly colored points representing the conjugate length seem to be concentrated in the high-OS areas. This implies that the conjugate length might correlate with the absorption and fluorescence wavelengths, along with the corresponding intensities, but the number of aromatic rings might not. To obtain more quantitative data, we computed the Pearson’s correlation coefficients listed in Table 1. The highest correlation (0.49) was between the conjugate length and the absorption wavelength and its coefficient, which supports the traditional prescription that designing molecules that absorb light with long wavelengths implies the elongation of the conjugate length (*32*).

**Table 1.** Pearson’s correlation coefficients (*R* values) of wavelengths and their OS of absorption to S_1_ states and fluorescence at the B3LYP/3-21G* level from S_1_ states with structural parameters (number of aromatic rings and conjugate length) of the generated molecules. See the Supplementary Materials for their correlation graphs (fig. S1).

	**S_1_ absorption**		**S_1_ fluorescence**	
	**Wavelength**	**OS**	**Wavelength**	**OS**
Number of aromatic rings	0.35	0.18	−0.04	0.13
Conjugate length	0.49	0.26	−0.03	0.24

To investigate the relationship between molecular features and the properties of absorption and fluorescence more fully, we developed prediction models on the basis of random forest (*33*) using the Mordred descriptors (*34*), as shown in table S1 and fig. S3; these models have been recently used in the field of cheminformatics. The absorption wavelength was found to be predictable to some extent (*R* = 0.73 whose SD was 0.028, evaluated by using fivefold cross validation), and features related to conjugation length appeared at the top as important features. On the other hand, it was difficult to predict the OS of absorption or the wavelength and OS of fluorescence, although a relationship between molecular structure and fluorescence in derivatives that include a common scaffold has been reported (*35*). All these analyses were only preliminary and did not exclude other molecular properties associated with fluorescence. Nevertheless, the fact that we could not find any correlation between fluorescence (or its OS) and molecular features, such as conjugate length and number of aromatic rings in the wide variety of molecules produced by ChemTS, illustrates the difficulty of designing fluorescent molecules by optimizing the molecular structure.

To select the candidate molecules for the synthesis, we imposed the following three conditions: (i) The OS of the molecule should be greater than 0.1 for S_1_ states from the S_0_ minimum. (ii) The molecule should emit fluorescence with a wavelength of over 400 nm and the OS of over 0.01. (iii) The difference between the wavelength of absorption and fluorescence in the S_1_ states should be greater than 100 nm to ensure distinguishability. Imposing (i) to (iii), we successfully filtered 87 molecules (table S2).

**Fig. 3.**
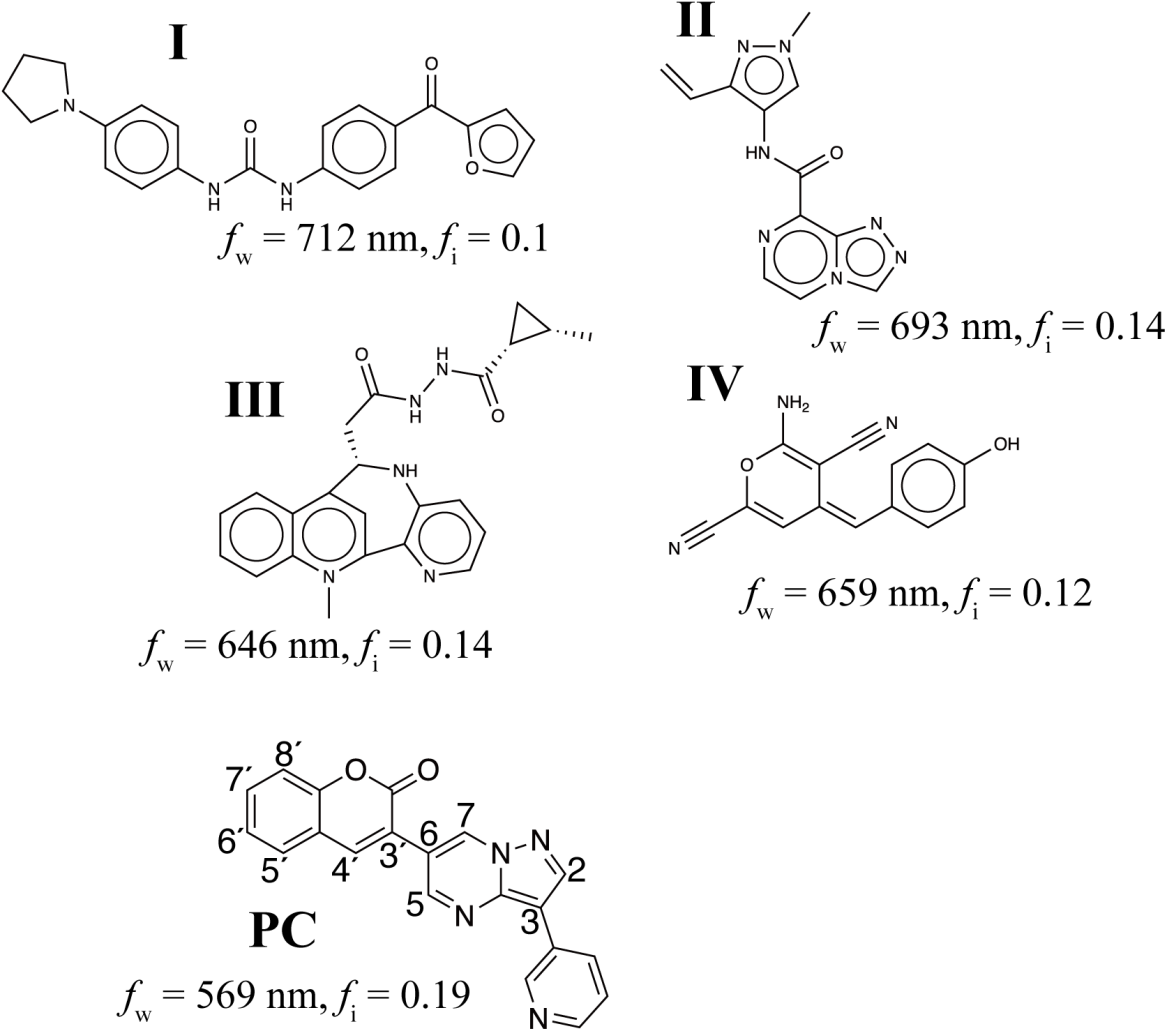
Unreported fluorescent molecules designed using ChemTS. Molecules I to IV are expected to emit near-infrared light. Molecule **PC** is 3-[3-(Pyridin-3-yl)pyrazolo[1,5-a]pyrimidin-6-yl]-2H-chromen-2-one, which was synthesized to validate in this work. The fluorescent light wavelength (*f*_w_) and corresponding OS (*f*_i_) were computed at the B3LYP/3-21G* level.

For comparison, we randomly selected 1000 molecules from the training dataset and evaluated their PESs to see whether they were computationally predicted to fluoresce. We found 661 computationally fluorescent molecules, four of which (constituting 0.61% of the training dataset) fluoresced with intensities detectable to the naked eye. By contrast, 87 of molecules generated by ChemTS (2.39% of all candidates) exhibited naked eye–detectable fluorescence. These molecules were relatively discovered in the second half of the run; the median order of generation of the 87 selected molecules was estimated to be 2035 (of 3643). Among them, we found seven known molecules or tautomers (see table S2) included in the chemical database SciFinder (*36*). We have experimentally confirmed that five of these seven molecules emit the visible fluorescence (see figs. S14 to S17 for their emission spectra and images and table S5 for the quantum yields). The other 80 molecules had not been reported yet; they include four fluorescent molecules that are predicted to emit light in the near-infrared region (around 700 nm) with moderate OS (Fig. 3) and might be useful for bioimaging molecules (*9*–*11*). None of these molecules have the common skeletons of known fluorescent molecules. This fact indicates the strength of ML; it can choose skeletons that synthetic chemists would not.

### Photochemistry of PC

To demonstrate the generator’s ability to discover novel compounds, we selected an unreported molecule that was not included in SciFinder (*36*). Because the molecules with novel π-aromatic frameworks are often difficult to synthesize, we chose our test case from the unreported coumarin derivatives, widely known as fluorescent molecules (*5*). The molecule that we selected, shown in Fig. 3, was 3-[3-(pyridin-3-yl)pyrazolo[1,5-a]pyrimidin-6-yl]-2*H*-chromen-2-one (**PC**).

This design principle of **PC** is a little bit strange from the professional viewpoint. Pyrazolopyrimidine, one of the groups in **PC**, has been used as a bioactive component (*31*) and has recently attracted as a fluorophore (*37*). Nevertheless, because of the way that the heterocycles are connected, **PC** is not a compound that would ordinarily attract the interest of chemists interested in synthesizing fluorescent materials. Although the substitution on the 3′ position of coumarin has been used to expand the π conjugation (*5*), a direct connection between two six-membered rings tends to induce a twisted structure (as in biphenyl derivatives) because of steric repulsion; this deformation breaks 𝜋 conjugation and shortens the absorption wavelength (*32*). Hence, the experts intuitively avoid the chromophore designs in which the six-membered ring of the pyrazolopyrimidine group directly connects to the 3′ position of coumarin (*5*). Unfavorable deformation can also be induced by the pyridinyl group connecting to the pyrazolopyrimidine group via its meta-position. We will return to the design principles of **PC** in a later subsection.

**Fig. 4.**
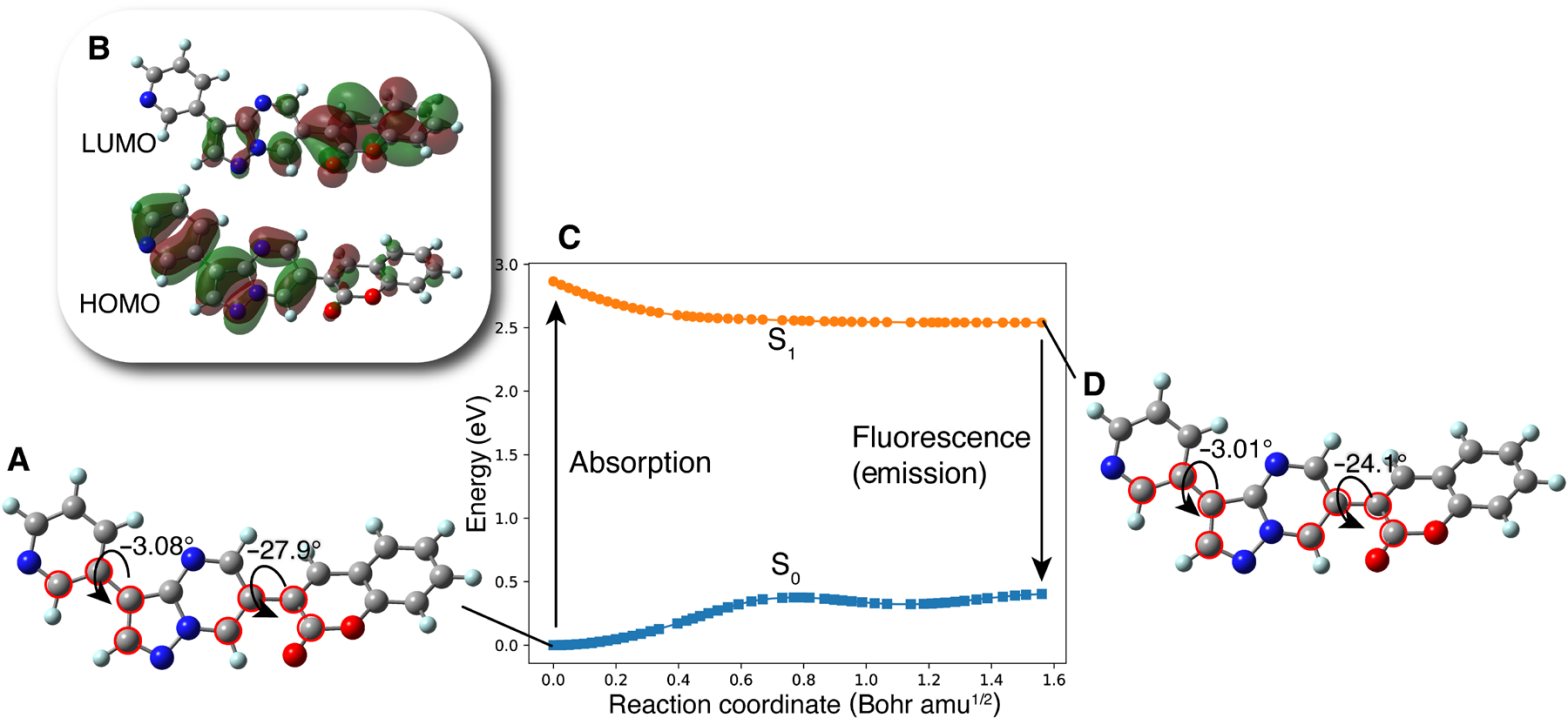
Photoinduced process of PC. (**A**) S_0_ minimum structure of **PC**. (**B**) The HOMO and the LUMO of optimized **PC** in the S_0_ state at the B3LYP/6-31G* level. The S_1_ state of **PC** results from the transition of one electron from HOMO to LUMO (𝜋-𝜋* excitation); the excitation (absorption) energy was computed to be 2.87 eV (433 nm) with OS of 0.192. The energy of fluorescence was computed to be 2.14 eV (580 nm) with OS of 0.128. (**C**) S_1_ minimum-energy path up to the S_1_ minimum structure. Energy is relative to S_0_ minimum. The nature of orbitals involved in the transition was not changed along the path. (**D**) S_1_ minimum structure of **PC**. The values of dihedral angles in (A) and (D) are measured from the carbon atoms marked by red circles.

Figure 4A shows the optimized structure of **PC** including its conformations (*38*) at the B3LYP/6-31G* level. In agreement with the arguments in the previous paragraph, **PC** had a slightly twisted structure. Its torsion angle, however, was no larger than we expected: The torsion angle between pyrazolopyrimidine and coumarin was estimated to be 27.9°. This is relatively small compared to that of 3-phenyl-coumarin, which has a torsion angle of 35.3°, according to our preliminary calculation at the B3LYP/6-31G* level; the Cartesian coordinate is shown in the Supplementary Materials. **PC**’s relatively small torsion angle is probably due to the weakness of the repulsion between 𝜋 orbitals of the rings. The orbitals of nitrogen-rich ring, pyrazolopyrimidine, have higher energies than that of hydrocarbon rings. The highest occupied molecular orbital (HOMO) of **PC** attributes to 𝜋 orbital of the pyridinyl-pyrazolopyrimidine moiety, as shown in Fig. 4B. In addition, the introduction of the nitrogen-rich ring results in charge transfer (CT) excitation. On the other hand, the torsion angle between pyridinyl and pyrazolopyrimidine is only 3.08°, reflecting the small steric repulsion induced by the pyridinyl group linked to the five-member ring of pyrazolopyrimidine at the meta-position.

According to the time-dependent (TD)–DFT computation, the vertical excitation to the S_1_ state from the S_0_ state of compound **PC** is due to the transition of the one electron from HOMO to the lowest unoccupied molecular orbital (LUMO) (𝜋-𝜋* excitation). As shown in Fig. 4B, the excitation to the S_1_ state induces a CT from the pyrazolopyrimidine moiety to the coumarin moiety. The S_1_ excited molecule reaches a stationary point in the S_1_ state with little increasing structural flatness, and this is enough to stabilize the S_1_ excited **PC** to over 0.33 eV from the S_1_ vertically excited point. We confirmed by the minimum energy path calculation that this minimum in the S_1_ state is accessible from the S_1_ vertically excited point without a barrier, as shown in Fig. 4C. Hence, **PC** is fluorescent, as was designed to be by ChemTS. Molecules in the excited state with the CT characteristic tend to show energetically large relaxations in their excited state, thereby causing moderate differences between the absorption and fluorescence; in the present case, the theoretically estimated difference between the two was 147 nm (S_1_ absorption wavelength, 433 nm; S_1_ fluorescence wavelength, 580 nm at the B3LYP/6-31G* level).

### Experimental measurements

**Fig. 5.**
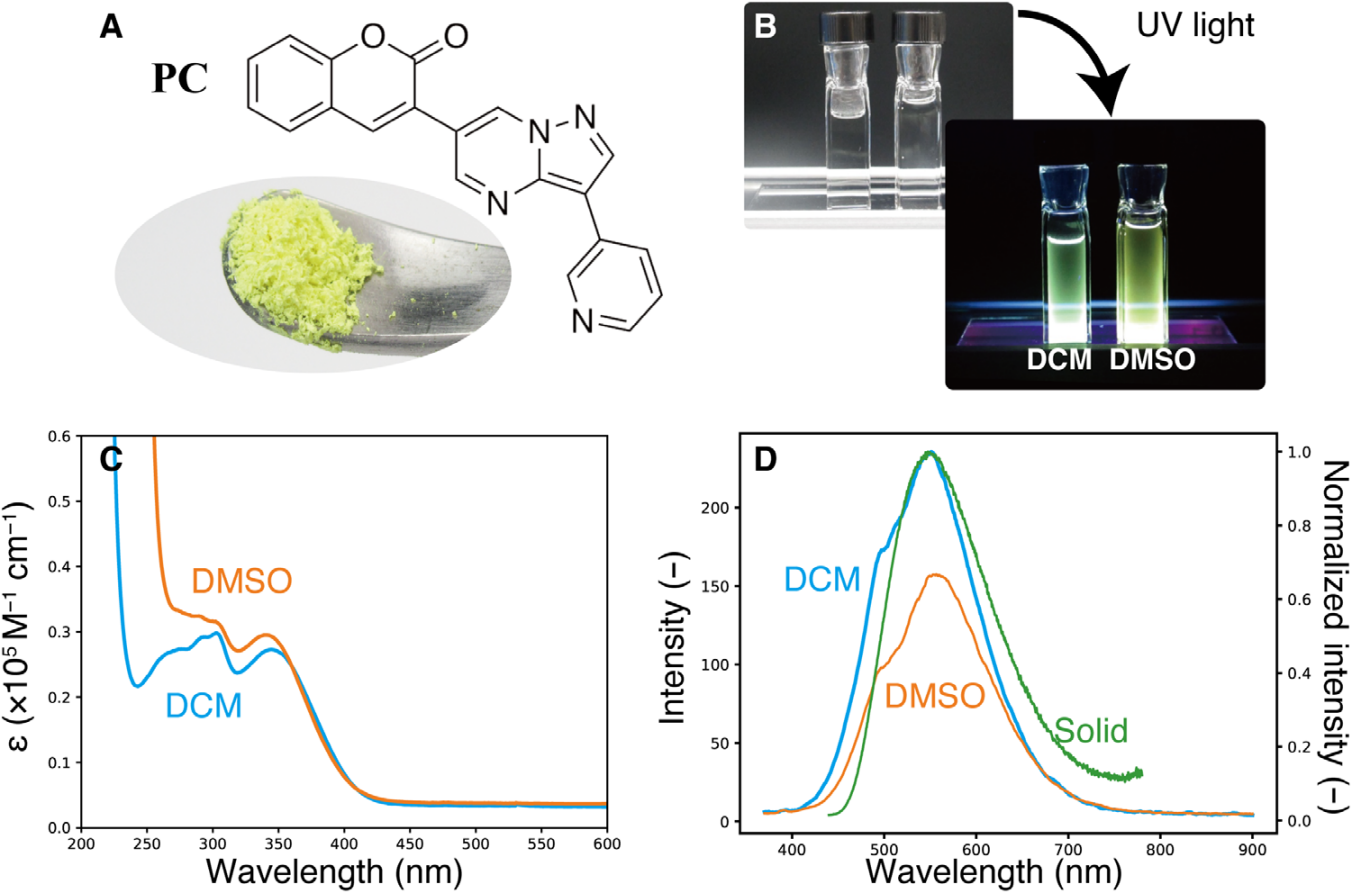
Photochemical properties of PC. (**A**) Image of **PC** in powdery form. (**B**) Images of **PC** dissolved in DCM and DMSO (100 μM) under room light and upon irradiation with UV light (365 nm). (**C**) UV-vis absorption spectra of **PC** in DCM and DMSO (10 μM; optical length, 1.0 mm; under air). (**D**) PL spectra (λ_ex_ = 350 nm) of **PC** in DCM, DMSO (10 μM; under air), and solid state. The intensity of the PL spectrum in solid state is normalized by its maximum value (right vertical axis).

Next, we describe validating the generator, synthesizing **PC**, and measuring its photoluminescence (PL) spectra. The synthesis route to **PC** and its characterization are described in the Supplementary Materials. Figure 5A shows that **PC** is a yellow solid material at room temperature. Figure 5B shows **PC** at a concentration of 0.1 mM in dichloromethane (DCM) and dimethyl sulfoxide (DMSO) solvents, first under room light and then upon the irradiation of UV light (365 nm). The fluorescence (yellow or green depending on the solvent) can be observed with the naked eye. The UV-vis absorption and PL spectra of **PC** in DCM and DMSO are shown in Fig. 5 (C and D, respectively). The PL spectrum of **PC** in the solid state resembled those of **PC** in the solutions, as shown in Fig. 5D. For more information on the concentration dependence of their spectra, see the Supplementary Materials. The UV-vis absorption spectrum of **PC** exhibits peaks with edges beginning at 425 nm, which is consistent with the theoretically predicted wavelength of the excitation energy to the S_1_ state. On the other hand, the first peak of the PL spectrum lies at around 550 nm, which is consistent with the theoretically predicted fluorescence from the S_1_ state. We could not find any increments in the intensity of PL when bubbled with dry N_2_ for 20 min; therefore, it can be concluded that phosphorescence does not contribute to the PL spectrum at room temperature (see the Supplementary Materials).

The experimental difference between the absorption and fluorescence spectra was evaluated to be approximately 125 nm, which is comparable to the theoretically predicted shift (147 nm). Although static theoretical computation succeeded in reproducing the experimental results, molecular dynamics did not. If the real crossing sites between the S_1_ and S_0_ PESs (conical intersections) were accessible by the molecular vibration in the S_1_ state (*39*, *40*), then S_1_ excited molecules could go back to their S_0_ states nonradiatively and the fluorescence yield would clearly decrease. This photoinduced dynamical behavior of the molecule in the S_1_ excited state was reflected in the value of the quantum yield (Φ); the Φ of **PC** measured by an integrating sphere in DCM was 0.007. This small Φ indicates that there were some nonradiative paths to the ground states (*39*, *40*). The S_2_ state is energetically close to the S_1_ state at the S_1_ minimum by 0.67 eV according to the DFT computation. Because a nonadiabatic decay route to the S_0_ state after 𝜋-𝜋* excitations of coumarin itself has been reported (*41*), there might be a nonradiative way to the S_0_ state after the internal conversion from the S_1_ state to the S_2_ state.

### Meaning of steric structure of PC

Although we succeeded in validating our fluorescent molecule generation system by synthesizing **PC** and measuring its fluorescence, we could not yet understand the design principle of **PC**. We therefore prepared isomers of **PC** and a coumarin derivative (**V**) that from standpoint of conventional design seemed likely to fluoresce on the basis of the computational results at the B3LYP/6-31G* level summarized in Table 2. We designed three **PC** isomers, differing in the connecting positions of pyridine to pyrazolopyrimidine and of pyrazolopyrimidine to coumarin. The steric repulsion was expected to be lower when coumarin was added at the 5-position of pyrazolopyrimidine (**PC2** and **PC3**) than when it was added at the 6-position (**PC** and **PC1**). In the case of **PC1** and **PC2**, the pyridine groups were linked with the pyrazolopyrimidine groups via their ortho-position to decrease the steric repulsion between them. Last, molecule **V** is a chromophore with the coumarin scaffold designed from the viewpoint of the structural planarity with a conjugated length similar to that of the **PC** species.

**Table 2.** PC species and a coumarin derivative V (a conventionally designed chromophore). Here, *a*_w_ and *a*_i_ are absorption wavelength and corresponding OS, and *f*_w_ and *f*_i_ are fluorescence wavelength and its OS, computed by the TD-DFT computation at the B3LYP/6-31G* level; θ_1_ and θ_2_ are torsion angles of each S_0_ optimized structure measured from atoms linked by bold type bonds.

**Species**	***a*_w_ (nm)**	***a*_i_**	***f*_w_ (nm)**	***f*_i_**	**θ_1_**	**θ_2_**
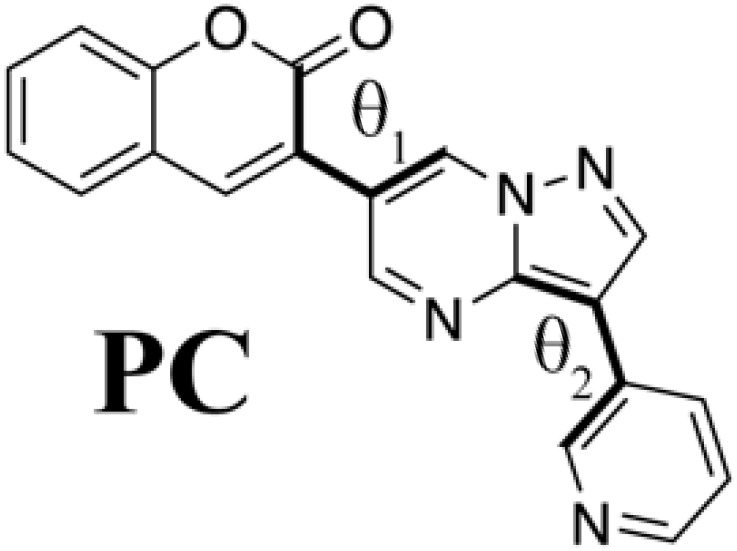	433	0.192	577	0.128	−27.9	−3.08
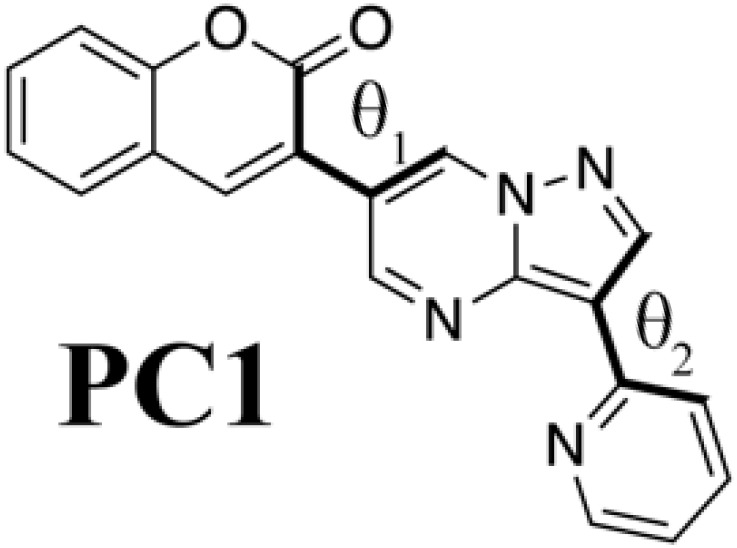	430	0.214	565	0.141	−28.2	−0.36
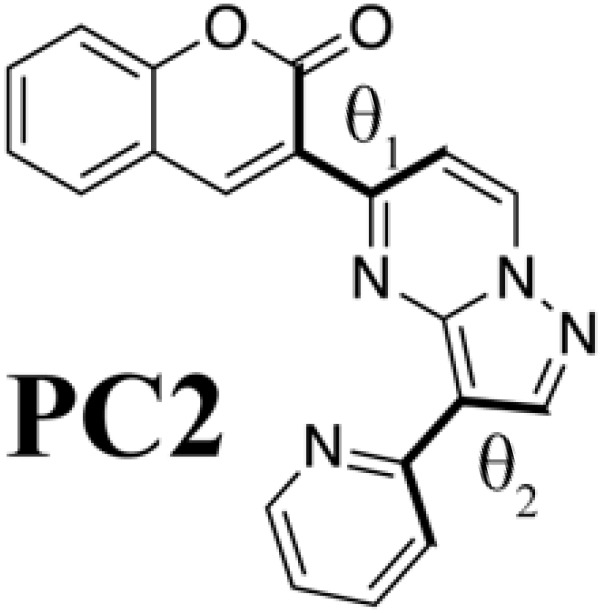	486.8	0.096	718	0.063	13.7	4.20
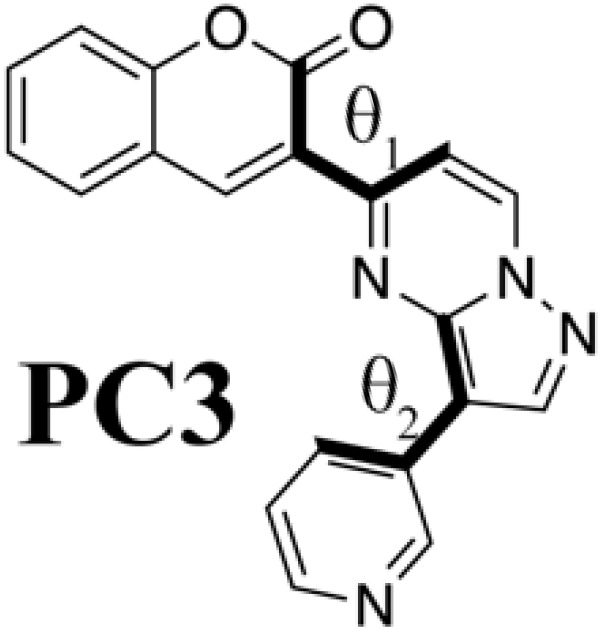	475	0.110	702	0.070	6.40	20.9
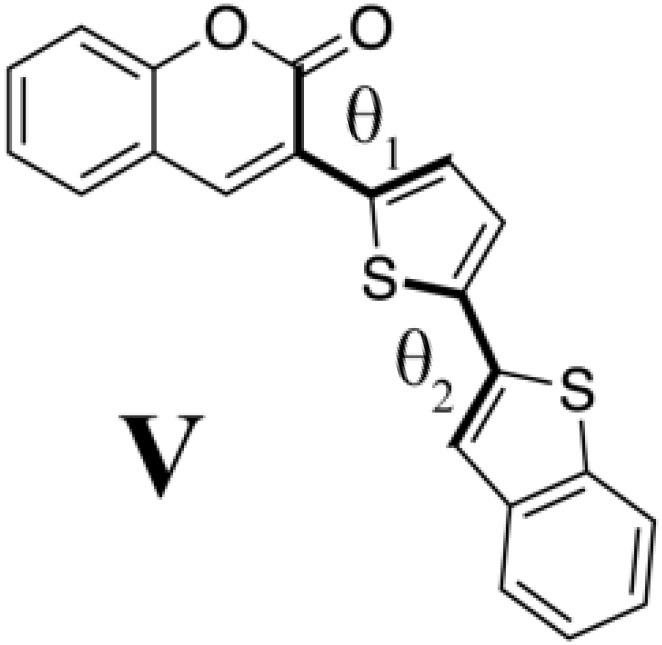	440	0.958	493	0.990	0.189	−0.802

Comparing **PC** itself with the other **PC** species, we can understand why **PC** has connections that increase the steric repulsion among coumarin, pyrazolopyrimidine, and pyridine groups. In the case of **PC2** and **PC3**, where the steric repulsion between pyrazolopyrimidine and coumarin is reduced [as reflected in the small torsion (θ_1_) angles between them], the wavelengths of fluorescence from the S_1_ state show a drastic red shift, while the OSs of absorption and fluorescence are decreased. For molecules that show thermally activated delayed fluorescence, increasing the steric repulsion around a bond is known to split the electronic distribution between HOMO and LUMO but to result in low OS (*4*). In the **PC** species, however, increasing the steric repulsion between pyrazolopyrimidine and coumarin increases their OS. Coumarin derivatives attached to the 7-position of pyrazolopyrimidine have already been reported as fluorophores (*37*) with large deformation from the flat plate (−37.4°). Although their intensity is high, their absorption (331 to 440 nm) and fluorescence (470 to 538 nm) show a slight blue shift in comparison with those of **PC** molecules (absorption, 425 nm; fluorescence, 550 nm). Therefore, **PC** is the result of ChemTS considering the trade-off between OS and wavelength.

On the other hand, the molecule **V**, designed from the professional viewpoint to fluoresce, certainly has a planar structure. The S_1_ excitation of **V** is a typical 𝜋-𝜋* excitation with high OS that induces the bond alternation. However, the fluorescence wavelength is not very long (493 nm), and the difference between it and the absorption wavelength (53.0 nm) is also small. These facts illustrate the difficulty of designing chromophores with the intention of absorbing and emitting target wavelength of lights.

## DISCUSSION

For several decades, QC has played an important role in chemistry and materials science. During this time, computer-aided molecular design has been used in drug discovery (*42*). However, QC has solely focused on the analysis and speculation of the experimental results; the creative work such as the prediction of various phenomena and designing materials (*6*, *43*–*47*) has rarely been considered. The recent applications of ML algorithms to chemistry and materials science (*48*, *49*) represent a positive turning point for computer-aided chemistry and materials science. To design molecules useful for organic electronics automatically, combining QC with a DNMG is crucial because, in such applications, quantum mechanics cannot be ignored. Nevertheless, the value of a DNMG based on QC must be proven before it can be adopted in practice.

In this study, we used a DNMG to create molecules with a property that cannot currently be easily predicted: fluorescence. We designed fluorescent compounds using DFT, an inherently quantum mechanical method. Although it is known that molecules are governed by the rules of quantum mechanics, it is difficult to create a new molecule de novo with only QC. Although fluorescent molecules have simple PESs, they are hard to design from first principles because their diversity makes it very difficult to correlate fluorescence with molecular structure.

However, the generator dealt with this diversity and succeeded in designing fluorescent molecules from scratch. The substantial ab initio computations based on QC required massive parallel computations (1024 cores, 5 days); nevertheless, the generator succeeded in producing 3643 candidate molecules. The generator produced molecules that absorbed light with long wavelengths, controlling the conjugate length of molecules in a manner similar to that of a professional; however, it could not find clear correlations between the fluorescence wavelength/intensity of molecules and the conjugate length/number of aromatic rings. This indicates the difficulties involved in the de novo design of fluorescent molecules.

We selected seven known compounds for validation and one candidate compound for further study on the basis of criteria of synthesizability and visible fluorescence. Experimental validation exhibited that the DNMG successfully designed fluorescent compounds with 75% (six of eight). The innovation potential of the DNMG was proven by the fluorescence (visible to the naked eye) of **PC**. In designing **PC**, the generator introduced an unfamiliar group, pyrazolopyrimidine, to coumarin; the connection induced a high steric repulsion but still resulted in an increase in OS. It is difficult for common chemists to come up with a way to enhance fluorescence and its intensity by increasing the steric repulsion between fragments. This shows that the generator is a tool that can surpass professional knowledge or intuition. DNMGs have the potential to induce a paradigm shift in molecular design.

Although the fluorescence of the molecule synthesized in this work was intended to be detected by the naked eye, more interesting molecules would be produced by removing the restrictions on the types of atoms and extending the design time. In addition, better molecular design would be possible by including the photoinduced dynamics of molecules. By further developing the QC, more complex functional molecules could be designed. Thus, generators with massively parallel computation would be able to create complex molecules with diverse and intriguing functions, possibly resulting in very complex synthesis routes that would increase the requirements of the recently developed planning retrosynthesis route based on ML (*50*–*52*).

## METHODS

### Molecular design

A simple fluorescent molecule has a minimum in the excited state with the same spin multiplicity as its ground state. Figure 1 shows an idealized PES of a molecule that emits fluorescence from its S_1_ state. Ignoring several factors that quench fluorescence, we designed the molecules with PESs similar to that shown in Fig. 1. Even in this simple framework, no qualitative guidelines are available for designing fluorescent molecules, owing to their diversity. A DNMG can address this. We used the molecule generator, ChemTS (*23*), which uses Monte Carlo tree search (MCTS) (*53*) and a recurrent neural network (RNN) (*54*, *55*) to generate molecules. Initially, the RNN was trained with a set of SMILES (simplified molecular-input line-entry system) strings (*56*). In our case, 153,253 molecules containing H, C, N, and O, obtained from the ZINC database (*57*), were used.

**Fig. 6.**
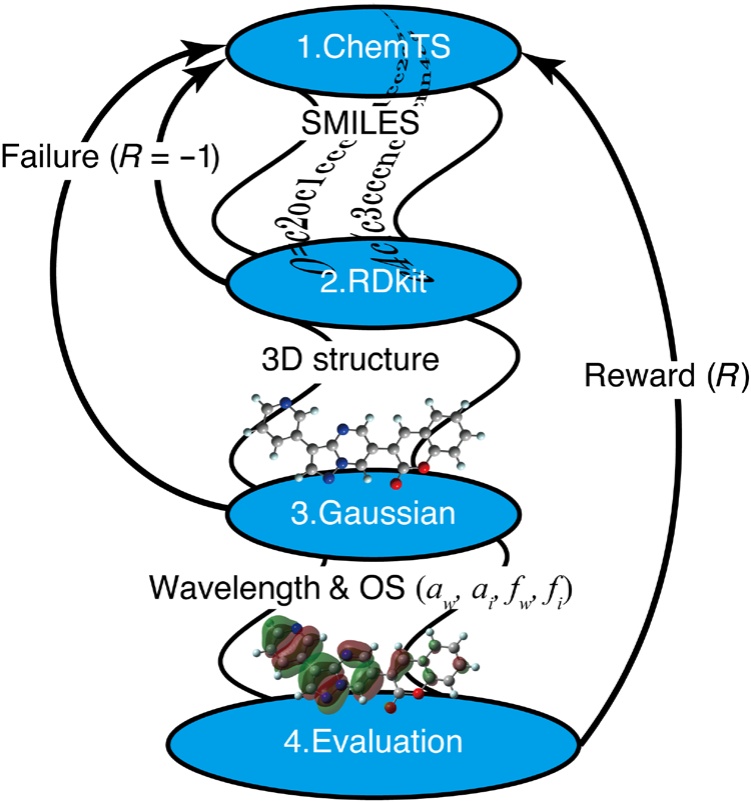
Workflow of fluorescent molecule generation. ChemTS generates the molecules represented in the SMILES strings (*56*), which are translated to a three-dimensional (3D) structure via RDKit (*58*). The absorption and fluorescence wavelengths and corresponding OSs obtained by the Gaussian package (*26*) are evaluated using the reward function (Eq. 1). ChemTS produces the molecules based on the electronic structure theory by iterating this process.

The PESs of the generated molecules were evaluated at the B3LYP/3-21G* level, implemented in the Gaussian 16 package (*26*). The excited energy and the fluorescence wavelength were computed by TD-DFT at the same level. The lowest 10 states of each molecule were calculated for each minimum in the S_0_ and S_1_ states. A flowchart illustrating the design of fluorescent molecules with ChemTS is shown in Fig. 6. To evaluate the absorption and fluorescence wavelengths and intensity simultaneously, we used the reward function

$$R(a_{w},a_{i},f_{w},f_{s})=W_{a_{w}}R_{a_{w}}(a_{w})+W_{a_{i}}R_{a_{i}}(a_{i})+W_{f_{w}}R_{f_{w}}(f_{w})+W_{f_{i}}R_{f_{s}}(f_{i})$$ (1)

Here, *W_a_w_, a_i_, f_w_, f_i__*, respectively, denote the weights of the reward functions *R_a_w_, a_i_, f_w_, f_s__*

$$R_{a_{w}}(a_{w})=\text{exp}(-\frac{{\left(a_{w}-T_{a_{w}}\right)}^{2}}{2\sigma_{a}^{2}})$$ (2)

$$R_{a_{i}}(a_{i})=\frac{\text{tanh}({\text{log}}_{10}(a_{i}+\epsilon )-{\text{log}}_{10}T_{a_{i}})}{2}$$ (3)

$$R_{f_{w}}(f_{w})=\text{exp}(-\frac{{\left(f_{w}-T_{f_{w}}\right)}^{2}}{2\sigma_{w}^{2}})$$ (4)

$$R_{\mathrm{fs}}(f_{s})=\frac{\text{tanh}({\text{log}}_{10}(f_{i}+\epsilon )-{\text{log}}_{10}T_{f_{i}})}{2}$$ (5)

*T_a_w__* and *T_a_i__* denote the upper absorption wavelength and its OS, respectively. Similarly, *T_f_w__* and *T_f_i__* are the upper fluorescence wavelength and its OS, respectively. In this work, we set *T_a_w__* and *T_f_w__* to 700 and 1200 nm, respectively, and their OS of *T_a_i__* and *T_f_i__* to 0.01. The values of *W_a_w__*, *W_a_i__*, *W_f_w__*, and *W_f_i__* were set to 0.4, 0.1, 0.4, and 0.1, respectively. We fixed values of σ*_a_*, σ*_f_*, and ϵ to 150, 150, and 10^−8^, respectively. The absorption and fluorescence wavelengths of the generated molecules are denoted by *a_w_* [with corresponding intensity (*a_i_*)] and *f_w_* [with corresponding intensity (*f_i_*)], respectively (see Fig. 1).

In this study, to accelerate the MCTS, we used a parallelization strategy based on the concept of virtual loss (*53*). When MCTS is parallelized, concentration of computation on a particular leaf node becomes a problem: If a subprocess on a leaf node has not finished its computation, then the same leaf node is selected by other subprocesses in the selection step. To solve this problem, parallelization with virtual loss explicitly treats whether a subprocess is computing on a node and uses the following score for each child node *i* in selection step

$$S_{i}=\frac{R_{i}}{v_{i}+w_{i}}+CP_{i}\sqrt{\frac{v_{p}+w_{p}}{1+v_{i}+w_{i}}}$$ (6)

If a subprocess is computing on a node, then the denominators increase, and that node will be less likely to be selected, resulting in distributed computation. Here, *R_i_* is the cumulative sum of the above reward *R*(*a_w_*, *a_i_*, *f_w_*, *f_i_*) of node *i*, *v_i_* is the total visit number of node *i*, *w_i_* is the total virtual visit number (virtual loss) of node *i*, *C* is the hyperparameter for search, and *P_i_* is the selection probability of node *i*; *v_p_* and *w_p_* denote the total number of visit and total virtual visit numbers of the parent node *p*, respectively. In this study, we set *C* to 4.

For geometric analysis, we selected the number of aromatic rings and the conjugate length. We counted aromatic rings using an RDKit function (*58*). For the conjugate length, we counted a unity that is the sequence of single bond–double bond–single bond (e.g., ─C═C─).

### Molecule synthesis and measurement

We selected one compound, **PC**, from those generated by ChemTS for chemical synthesis using ease of synthesis as a criterion. Because the computational level used for molecular design was B3LYP/3-21G*, which is somewhat unreliable, the PES of **PC** was checked at the B3LYP/6-31G* level before synthesis. In addition to **PC**, we synthesized seven molecules reported in SciFinder (*36*) for analyzing absorption and emission spectra. Compound **PC** and the seven other compounds were obtained from Tokyo Chemical Industry Co. Ltd. through custom synthesis (refer to the Supplementary Materials for the detailed synthesis process and its characterization of **PC**).

The electronic absorption spectra were measured at 20°C using a Shimadzu UV-3600 UV–vis–near-infrared spectrophotometer. The emission spectra in solution and solid state were measured using FP-8300 (JASCO, Japan). A quartz cell with an optical length of 1.0 mm or 1.0 cm was used. Spectroscopic-grade solvents (DCM and DMSO) were obtained from FUJIFILM Wako Pure Chemical Corporation. Absolute fluorescence quantum yields were determined with a Hamamatsu Photonics C-9920-02 calibrated integrating sphere system.

## Supplementary Materials

### This PDF file includes:

Sections S1 to S7 Figs. S1 to S41 Tables S1 to S5
