# Supplementary material for: De novo creation of a naked eye–detectable fluorescent molecule based on quantum chemical computation and machine learning
Source: Sci Adv. 2022 Mar 9;8(10):eabj3906. doi: 10.1126/sciadv.abj3906 (PMC8906732; doi:10.1126/sciadv.abj3906)
Supplement: Supplementary file 1 — Sections S1 to S7 Figs. S1 to S41 Tables S1 to S5 [file sciadv.abj3906_sm.pdf]

Science Advances
AAAS

# Supplementary Materials for

## De novo creation of a naked eye–detectable fluorescent molecule based on quantum chemical computation and machine learning

Masato Sumita*, Kei Terayama, Naoya Suzuki, Shinsuke Ishihara, Ryo Tamura, Mandeep K. Chahal, Daniel T. Payne, Kazuki Yoshizoe, Koji Tsuda*

*Corresponding author. Email: masato.sumita@riken.jp (M.S.); tsuda@k.u-tokyo.ac.jp (K.T.)

Published 9 March 2022, *Sci. Adv.* **8**, eabj3906 (2022)
DOI: 10.1126/sciadv.abj3906

**This PDF file includes:**

Sections S1 to S7
Figs. S1 to S41
Tables S1 to S5

# 1. Correlation with number of aromatic rings

To see the correlations of absorption wavelength to $S_1$ states/emission wavelength from $S_1$ states and its oscillator strengths (OSs) with the number of aromatic rings, the correlation graphs with them are shown in Figure S1. We used the tools of RDkit (59) to count aromatic rings.

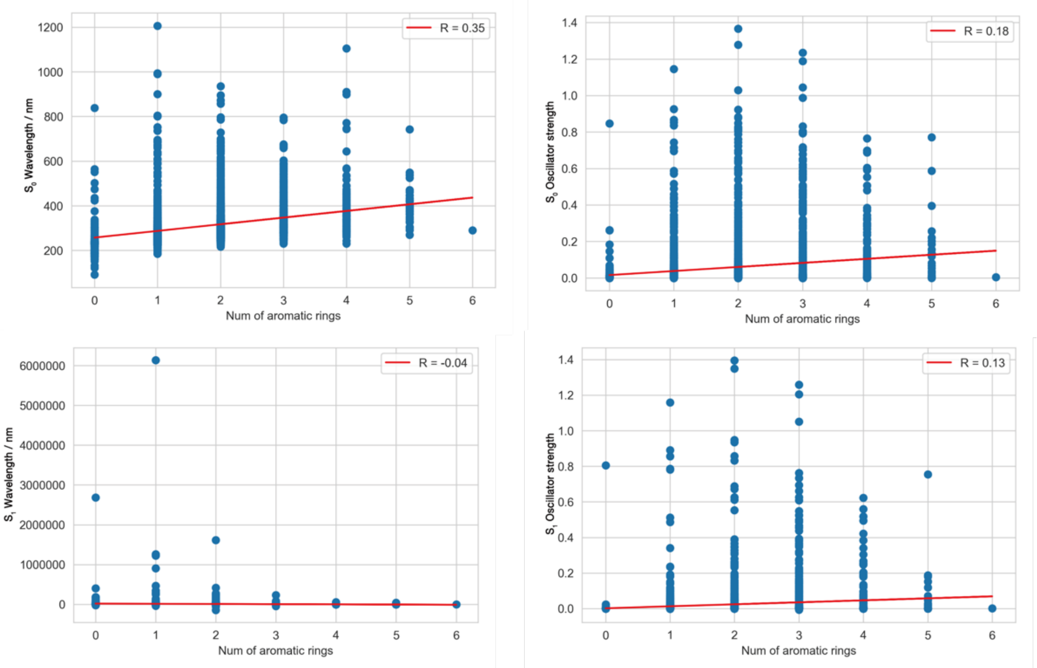


**Figure S1. Correlation graphs of fluorescence/absorption wavelength and their intensities with aromatic rings.** Upper two are correlation graphs of number of aromatic rings with $S_0$ absorption wavelength, its Oscillator strength. Bottom two are correlation graphs with $S_1$ emission wavelength, its oscillator strength (bottom two).

## 2. Correlation with conjugate length

To see the correlations of absorption wavelength to $S_1$ states/emission wavelength from $S_1$ states and its oscillator strengths (OSs) with conjugate length, the correlation graphs with them are shown in Figure S1. We have count conjugate length whose unity is defined as single-double-single bond sequence.

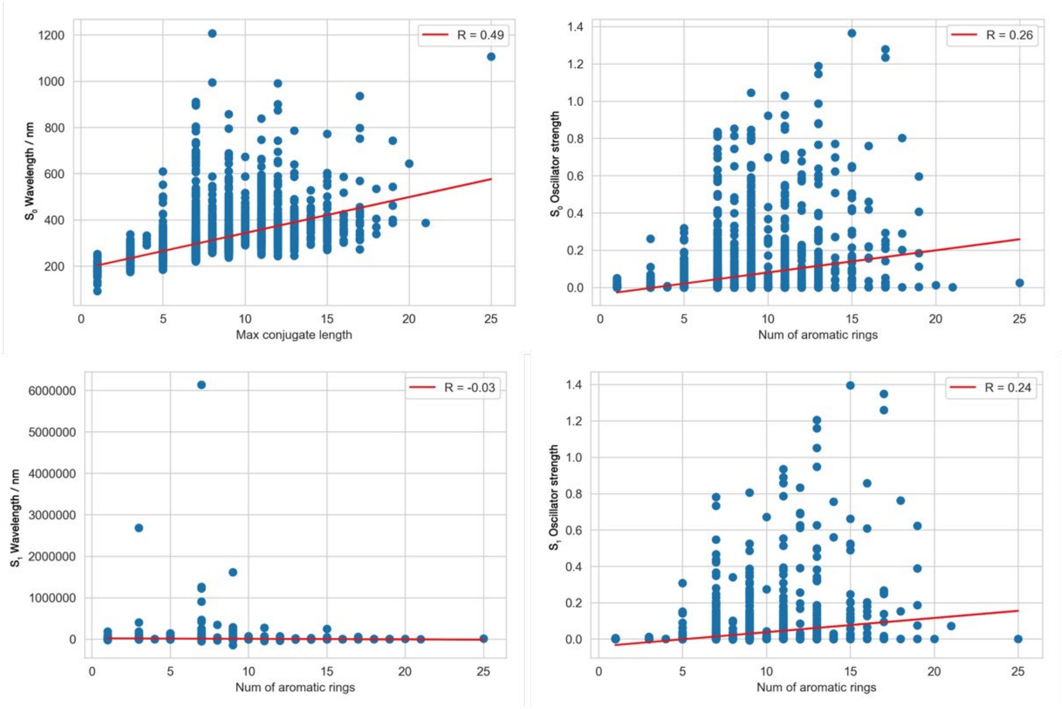


**Figure S2. Correlation graphs of fluorescence/absorption wavelength and their intensities with conjugate rings.** Upper two are correlation graphs of number of conjugate lengths with $S_0$ absorption wavelength, its Oscillator strength. Bottom two are correlation graph with $S_1$ emission wavelength, its oscillator strength.

## 3. Prediction of absorption and emission by machine learning model

For investigation of the relationship between molecular features and the properties of absorption and fluorescence, we developed prediction models based on random forest using the Mordred descriptors. We employed an implementation of the random forest regression model in scikit-learn library. The number of trees was set to 100, and other settings were used as default. The prediction performance was evaluated using 5-fold cross validation. The average of correlations of the prediction results for each property is shown in Table S1. The features with high feature importance in each prediction model are shown in Figure S3.

**Table S1**. Pearson's correlation coefficients (R values) of wavelengths and their intensities of absorption to $S_1$ states and fluorescence at the B3LYP/3-21G* level from $S_1$ states with the predicted values by the trained random forest models. Standard deviations (SDs) of the R values are shown in parentheses. Mean absolute error (MAE) values are also given.

| | Wavelength | | OS | |
|---|---|---|---|---|
| | R | MAE / nm | R | MAE |
| $S_1$ absorption | 0.73 (0.028) | 35.4 | 0.35 (0.074) | 0.0653 |
| $S_1$ fluorescence | 0.19 (0.040) | 428 | 0.27 (0.036) | 0.0402 |

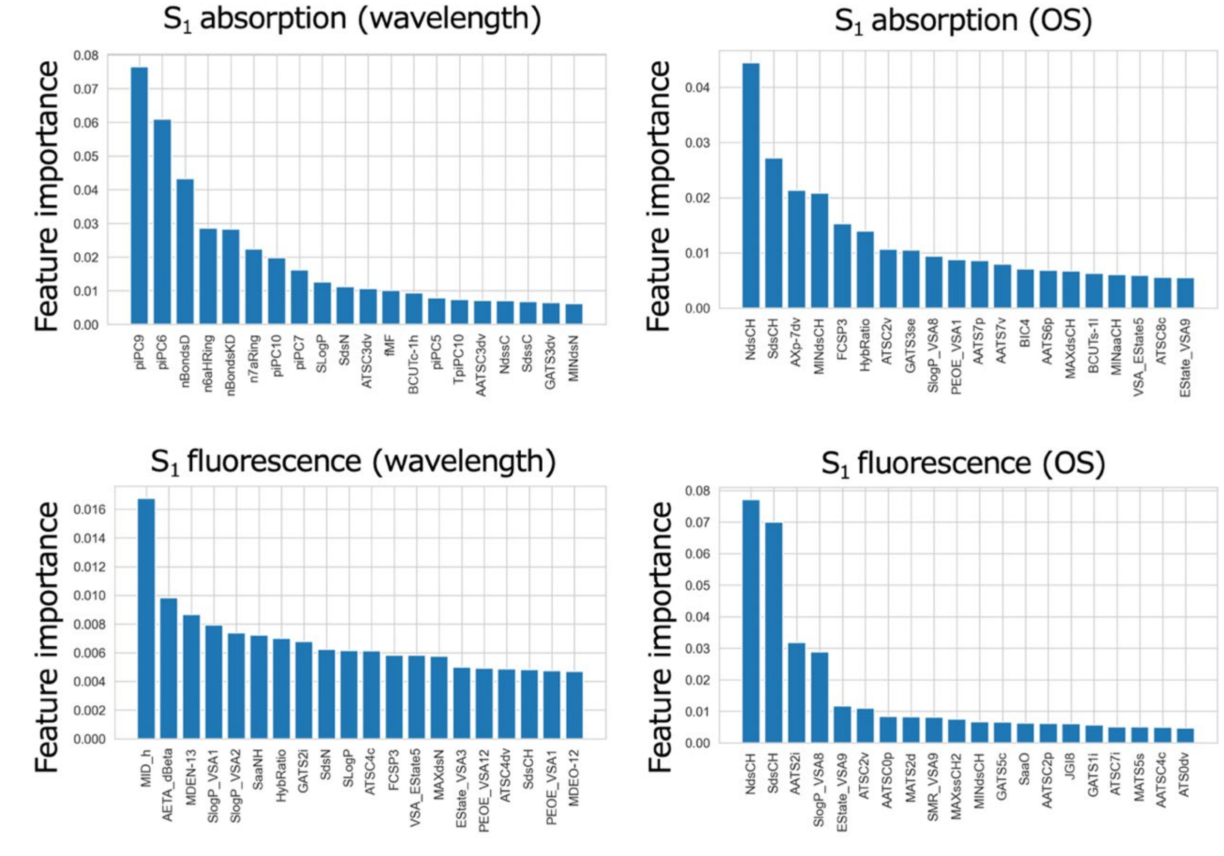


**Figure S3. Top 20 highest features in each prediction model.** The names of the features correspond to the descriptions in Mordred (34).

# 3. Selected molecules

From the viewpoint of detectability, we have selected 87 molecules in accordance with the condition mentioned in the main text. In Table S2, selected 87 molecules in SMILES string are summarized with their properties computed at the B3LYP/3-21G* level. **A-G** are molecules or its tautomers that are found in Scifinder and synthesized for the experimental validation. The unreported molecule we synthesize in this study is **PC**. **I-IV** are expected to emit near-infrared light.

**Table S2.** Selected 87 molecules and their properties from the viewpoint of detectability

| Species in SMILES | $S_0$ absorption /nm | $S_0$ OS | $S_1$ emission /nm | $S_1$ OS |
|---|---|---|---|---|
| Nc1nonc1C(=O)Nc1ccc2[nH]ncc2c1 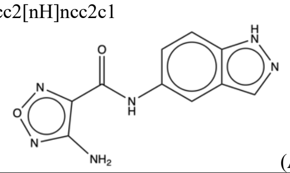 **(A)** | 362 | 0.20 | 533 | 0.15 |
| Cn1c(C(=O)Nc2cccc(F)c2F)nc2ccccc21 | 305 | 0.54 | 446 | 0.38 |
| N/C=C/c1c(O)cccc1O | 269 | 0.25 | 516 | 0.04 |
| Nc1n[nH]nc1-c1cccnc1 | 294 | 0.15 | 497 | 0.01 |
| Cn1cc(/C=N/n2nnc3ccccc32)c2ccccc12 | 339 | 0.55 | 447 | 0.30 |
| Nc1c(-c2n[nH]c3ccccc23)cccc1C(F)(F)F | 352 | 0.17 | 459 | 0.06 |
| C/C(=C\C(=O)c1cnoc1C)C(=O)Nc1ccc2nc(C)ccc2c1 | 410 | 0.20 | 565 | 0.27 |
| O=c1n2nc(-c3ccccn3)oc2cc2cc(N3CCCC3)cc(C(F)(F)F)c12 | 409 | 0.12 | 655 | 0.03 |
| O=c1[nH]cc(/C=C/c2ccc(O)nc2)c2c1CCCCC2 | 325 | 0.49 | 440 | 0.02 |
| C/C(=C\c1ccc(C(C)C)cc1)O[C@@H](C)C1CC1 | 253 | 0.32 | 1238 | 0.03 |
| CN1N=C(C(=O)Nc2cccc(NC(=O)c3ccco3)c2)CC1=O | 358 | 0.13 | 600 | 0.05 |
| CNc1nc(-c2c(F)cccc2F)ccc1C(=O)N1CCCCC1 | 382 | 0.18 | 505 | 0.08 |
| NN[C@H](CCC1=c2ccccc2=NC1)C(=O)Nc1ccccc1F | 378 | 0.13 | 770 | 0.02 |
| O=C(c1ccc(NC(=O)Nc2ccc(N3CCCC3)cc2)cc1)c1ccco1 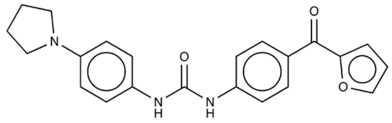 **(I)** | 460 | 0.13 | 712 | 0.10 |
| Cc1cc(C)c(C#N)c(=O)n1Cc1noc(C)n1 | 307 | 0.19 | 453 | 0.02 |
| O=c1occ2c3ccccc3nc2cc1C | 360 | 0.17 | 550 | 0.06 |
| CCn1cc(/C=C2/C(=O)Nc3ccc(C)cc32)c2c(O)nn(C)c21 | 379 | 0.23 | 643 | 0.03 |
| O=C1N(CCC(F)(F)F)c2c(ccn2-c2ccc(F)cc2)/C1=C\c1cccc(F)c1 | 434 | 0.15 | 841 | 0.03 |
| Fc1cn(CN2CCCC[C@@H]2CO)nc1/N=C1\C=C(c2ccccn2)OC1 | 368 | 0.64 | 3874 | 0.02 |
| O=c1oc2ccccc2cc1-c1cnc2c(-c3cccnc3)cnn2c1 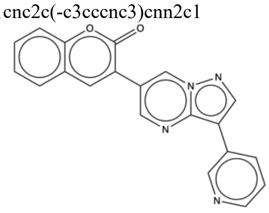 **(PC)** | 434 | 0.26 | 569 | 0.19 |
| Fc1ccc(-c2ccccc2Nc2ncnn2Cc2ccncc2)o1 | 309 | 0.16 | 472 | 0.06 |
| C=Cc1nn(C)cc1NC(=O)c1nccn2cnnc12 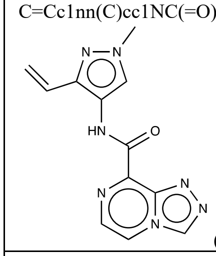 **(II)** | 510 | 0.13 | 693 | 0.14 |
| Fc1cc2ccnn2c(-c2ccccc2)n1 | 347 | 0.17 | 474 | 0.10 |
| Cn1c2cc([C@H](CC(=O)NNC(=O)[C@@H]3C[C@@H]3C)nc3cccnc23)c2ccccc21 | 455 | 0.23 | 646 | 0.14 |

| | | | | |
|---|---|---|---|---|
| 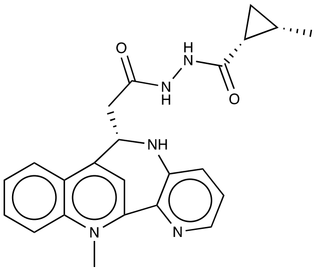 (**III**) | | | | |
| O=c1nc(NCc2cccc(C(F)(F)F)c2)cnn1-c1ccc(F)cc1 | 348 | 0.31 | 538 | 0.16 |
| C#Cc1ncc2c3ccccc3n(CC(=O)N3CC[C@H](C)C[C@H]3c3ccccc3)cc12 | 440 | 0.16 | 752 | 0.05 |
| CCn1c(C)nc2cc(NC(=O)c3cc(C)no3)ccc21 | 343 | 0.32 | 473 | 0.35 |
| CCO[C@@H]1C[C@@H](NC(=O)c2ccc(-n3cc(C(=O)NC(C)C)ccc3=O)cc2)C12CCCCC2 | 321 | 0.23 | 457 | 0.03 |
| C/C(=C\NC(=O)c1ccco1)c1cccc(C#N)c1 | 324 | 0.59 | 523 | 0.34 |
| COc1nn(CC)cc1CNc1ccc(-c2ccccc2)cc1 | 289 | 0.61 | 571 | 0.01 |
| Nc1n[nH]nc1-c1ccc(C(=O)NC2CC2)cc1 | 306 | 0.29 | 455 | 0.02 |
| COc1ccc(/C=C(/C#N)C(=O)N2CCCC2)cc1 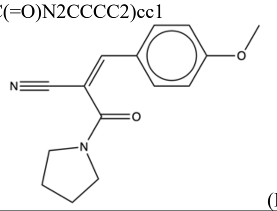 (**B**) | 325 | 0.26 | 663 | 0.01 |
| Nc1cc(NC(=O)c2ccc3c(c2)OCCO3)nn1-c1cccnc1 | 309 | 0.20 | 494 | 0.09 |
| N#C/C1=C/C(=C/c2ccc(O)cc2)C(C#N)=C(N)O1 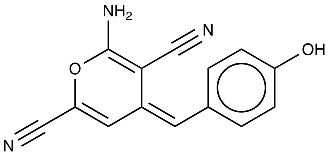 (**IV**) | 427 | 0.21 | 659 | 0.12 |
| CC[C@H](CCO)CNc1cnn(-c2ccc(F)cc2)c(=O)c1C | 318 | 0.22 | 472 | 0.18 |
| CCc1c(/C=C/c2cccnc2)[C@H](C(=O)O)[C@H](C)N=C1N | 383 | 0.12 | 754 | 0.02 |
| Cc1nc(N2CCN(C[C@@H]3CCCCN3CCC)CC2)c2nc[nH]c2c1 | 280 | 0.20 | 598 | 0.02 |
| O=C(N/N=C/c1ccccn1)c1nc(C2CC2)n2ccccc12 | 347 | 0.25 | 459 | 0.09 |
| O=C([C@H]1Cc2ccccc2CN1C(=O)C1=NN(c2ccccc2)C(=O)CC1)N1CCOCC1 | 323 | 0.12 | 874 | 0.02 |
| O=c1c(-c2nc(C3CC3)no2)cc(O)c2ccn(-c3ccc(F)cc3)nc12 | 595 | 0.24 | 913 | 0.07 |
| C#CCN(CC#C)C(=O)c1cccnc1Nc1ccc(C)cc1 | 404 | 0.11 | 716 | 0.04 |
| O=C(N/N=C/c1cnn(-c2ccccc2)c1)c1ccccc1N | 320 | 0.79 | 793 | 0.05 |
| NC(=O)[C@H]1N=c2ccccc2=C1NC(=O)c1ccco1 | 463 | 0.24 | 921 | 0.04 |
| C/C(=N\Nc1cccc2nnc(C)nc21)c1ccc(O)cc1 | 509 | 0.14 | 782 | 0.08 |
| Fc1cc2c(-c3ccccc3)nc3c(-c4ccc(F)cc4)cnn3c2cc1F | 426 | 0.22 | 601 | 0.15 |
| Nc1c(-c2ccccc2)[nH]c2c(C(F)(F)F)ccnc12 | 420 | 0.10 | 662 | 0.03 |
| NC(=O)[C@H]1N=C(c2ccc(F)cc2)CO1 | 317 | 0.11 | 544 | 0.04 |
| N#C/C1=C(c2ccccn2)Nc2nc3ccccc3n2C1 | 374 | 0.11 | 501 | 0.14 |
| O=C(Cc1ccco1)NCCCn1cnc2c(cnn2-c2ccccc2)c1=O | 287 | 0.34 | 403 | 0.19 |
| Nc1ncnc(Nc2ccccc2C#N)c1[C@@H]1CCCO1 | 312 | 0.15 | 431 | 0.03 |
| O=c1[nH]c(Oc2cccc(F)c2)c2ccccc2c1C(F)(F)F | 410 | 0.12 | 537 | 0.08 |
| O=c1[nH]cnc(Oc2ccc(F)cc2)c1C(F)(F)F | 313 | 0.15 | 566 | 0.05 |
| O=c1c(-c2ccccc2)cc(NCC(=O)N(c2ccccc2)C2CCCC2)co1 | 389 | 0.13 | 496 | 0.11 |
| CC[C@@H]1c2ccccc2C(=O)N1Cc1nc(-c2ccc(N)cc2)no1 | 303 | 0.23 | 507 | 0.02 |
| O=c1[nH]cccc1C(=O)Nc1ccc(F)cc1 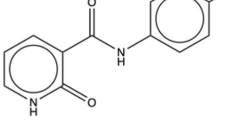 (**C**) | 387 | 0.15 | 565 | 0.16 |
| CNc1ncccc1C(=O)Nc1cccc2cccnc12 | 376 | 0.12 | 578 | 0.05 |

| | | | | |
|---|---|---|---|---|
| 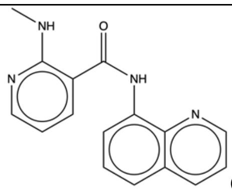 (**D**) | | | | |
| O=c1ccc(-c2ccccc2)nc2c1-c1ccccc1-2 | 587 | 0.16 | 986 | 0.06 |
| N#CCn1cc(Nc2ncnc3c2cnn3-c2ccccc2F)c(-c2ccco2)c1 | 328 | 0.39 | 473 | 0.12 |
| O=c1[nH]c(-c2ccccc2)nc2ccccc12 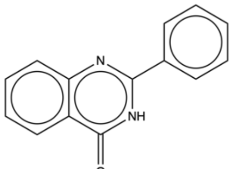 (**E**) | 303 | 0.42 | 523 | 0.13 |
| O=c1[nH]c(O[C@H]2COCCO2)c2ccccc2c1C(F)(F)F | 410 | 0.11 | 542 | 0.06 |
| O=c1n(c2ccc3c(c2)OCCCO3)nc[nH]c1=O | 347 | 0.16 | 479 | 0.08 |
| O=C(/C=C/C1CCCC1)NNC(=O)c1ccc(=O)n(-c2ccccc2)n1 | 351 | 0.28 | 464 | 0.20 |
| N/C(=N/Nc1cnc2ccc(F)cc2n1)c1ccc2ccccc2c1 | 420 | 0.19 | 595 | 0.04 |
| Cc1nn2c(nnc3c(=O)n(Cc4ccc(F)cc4)cnc32)c1-c1ccc(F)cc1 | 470 | 0.14 | 817 | 0.06 |
| COc1ccc(-n2nc(C)c(CNC(=O)[C@H]3CCCN3C(=O)[C@@H](C)NC)c2C)cc1 | 257 | 0.11 | 481 | 0.12 |
| O=c1[nH]cccc1-c1ccc(C(F)(F)F)cc1 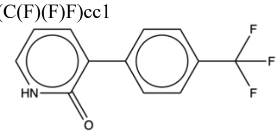 (**F**) | 322 | 0.36 | 432 | 0.06 |
| O=C(/C=C/c1cc(O)ccc1O)Nc1cccc2ccccc12 | 368 | 0.46 | 529 | 0.39 |
| Fc1cc2nc3c(nn2c1)CCN(Cc1ccco1)CN3 | 381 | 0.10 | 554 | 0.03 |
| N#C/C(=C/c1ccc(C(F)(F)F)cc1)c1nc2ccccc2c(=O)[nH]1 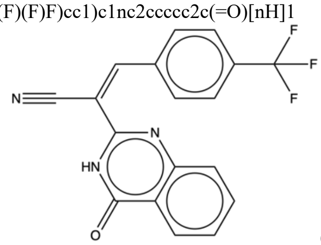 (**G**) | 370 | 0.29 | 559 | 0.15 |
| Fc1c(/N=C/c2coc3ccccc3c2=O)ccc2c1C(=O)c1ccccc1-2 | 415 | 0.29 | 521 | 0.25 |
| Nc1[nH]c(CN2CCCC2)c(C(=O)NC[C@@H]2CCCO2)c1N | 280 | 0.11 | 540 | 0.03 |
| Nc1ccc(-c2cc(C(=O)O)ncn2)cc1 | 362 | 0.21 | 469 | 0.08 |
| O[C@H](CCC1=c2ccccc2=NC1)c1cnn(-c2ccccc2)c1 | 377 | 0.14 | 760 | 0.02 |
| Fc1cc2c3nc(CN4CCCC4)cc(-c4ccccc4)n3nc2cc1F | 401 | 0.11 | 572 | 0.07 |
| O=C(N/N=C/C1=c2ccccc2=NC1)N1CCN(C(=O)C2CCCCC2)CC1 | 436 | 0.37 | 682 | 0.10 |
| O=c1c(O[C@@H]2C[C@H]3CC[C@@H]2C3)c(-c2ccccc2)[nH]1 | 436 | 0.11 | 1075 | 0.04 |
| COCc1ccccc1NC(=O)Nc1ccc(N(C)C(C)=O)cc1 | 285 | 0.26 | 540 | 0.01 |
| O=c1[nH]cnc(N2C[C@H]3CCCCC[C@@H]3C2)c1-c1ccccc1 | 321 | 0.20 | 463 | 0.15 |
| O=C(O)c1cccc(C(=O)Nc2cccnc2F)c1 | 298 | 0.11 | 452 | 0.10 |
| Cc1ccc(/C=N/c2n(C)c(=O)c3c(F)c(F)cc(F)c3n2)c(F)c1 | 389 | 0.41 | 521 | 0.39 |
| N/C(N/N=C/c1nc(-c2ccccc2)nc2cccn12)c1c[nH]c2ccccc12 | 422 | 0.12 | 576 | 0.07 |
| O=c1[nH]c(/C=N\c2nnc(-c3ccccc3)o2)cn1C | 400 | 0.65 | 777 | 0.11 |
| Nc1nn(/NCc2ccccc2)c(=O)c2cn[nH]c12 | 342 | 0.12 | 617 | 0.03 |
| O[C@H](CCC1=c2ccccc2=NC1)c1ccc(F)cc1F | 376 | 0.13 | 815 | 0.02 |
| COc1cc(-c2nc3c(C)cccc3c(=O)[nH]2)c2c(C(F)F)cc(=O)n(Cc3ccccc3)c2c1 | 350 | 0.16 | 466 | 0.18 |
| O=c1n2cnc(-c3ccccc3F)nc2cc(N2CCc3ccccc3C2)c1-c1ccc(F)cc1 | 462 | 0.18 | 610 | 0.19 |
| O=C(c1c[nH]nc1-c1ccc2c(c1)OCO2)N1CCOC2(CCCCC2)C1 | 285 | 0.16 | 441 | 0.07 |

# 4. Experimental Details of PC

## 4.1. Materials

3-[3-(Pyridin-3-yl)pyrazolo[1,5-a]pyrimidin-6-yl]-2H-chromen-2-one (**PC**) was obtained from Tokyo Chemical Industry Co., Ltd. through custom synthesis. Spectroscopic grade solvents ($CH_2Cl_2$ and DMSO) were obtained from Fujifilm Wako Pure Chemical Corporation.

## 4.2. General methods

ATR-FTIR spectra were obtained using a Thermo-Nicolet 760X FTIR spectrophotometer equipped with a SMART-iTX ATR accessory. $^1$H-NMR spectra were obtained using a JEOL JNM-ECA400 spectrometer operating at 400 MHz and using tetramethylsilane (TMS) as an internal standard. Proton decoupled $^{13}$C-NMR spectra were obtained using a JEOL JNM-ECA400 spectrometer operating at 101 MHz and using TMS as an internal standard. Data was processed using Delta version 5.0.5.1. $^1$H NMR chemical shifts (δ) are reported in ppm relative to TMS in DMSO-$d_6$ (δ = 0.00). $^{13}$C NMR chemical shifts (δ) are reported in ppm relative to the solvent reported. Coupling constants (*J*) are expressed in Hertz (Hz), shift multiplicities are reported as singlet (s), doublet (d), triplet (t), quartet (q), double doublet (dd), multiplet (m) and broad singlet (bs). High resolution ESI-MS mass spectra were measured using a Thermo Scientific Q-Exactive Plus instrument in methanol with 0.1% formic acid.

## 4.3. Synthesis

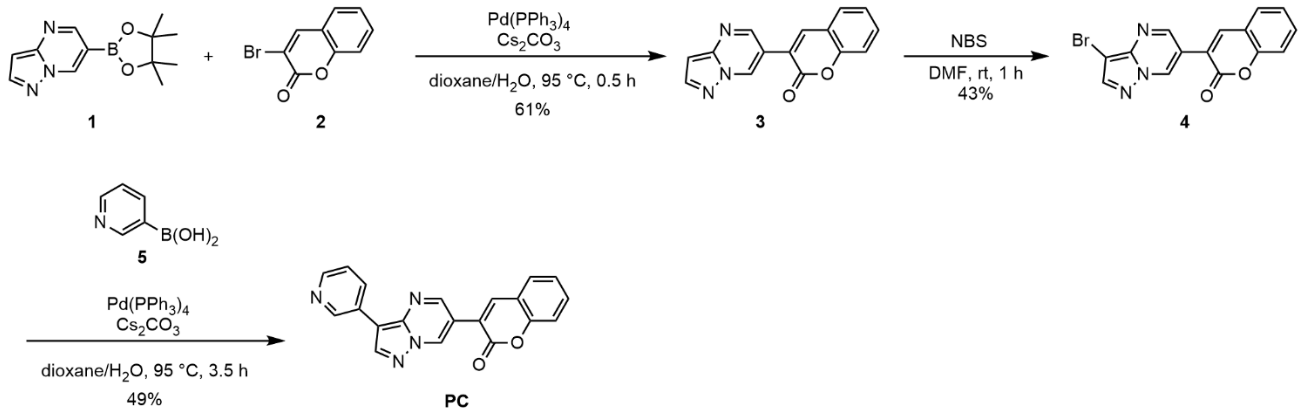


**Figure S4. Synthesis of 3-[3-(Pyridin-3-yl)pyrazolo[1,5-a]pyrimidin-6-yl]-2H-chromen-2-one (PC).**

Synthesis of **3**. 6-(4,4,5,5-Tetramethyl-1,3,2-dioxaborolan-2-yl)pyrazolo[1,5-a]pyrimidine (**1**) (0.80 g, 3.3 mmol), 3-bromo-2-chromenone (**2**) (0.74 g, 3.3 mmol), tetrakis(triphenylphosphine)palladium(0) (188 mg, 0.163 mmol), and $Cs_2CO_3$ (3.2 g, 9.8 mmol) were dispersed in a mixture of 1,4-dioxane (16 mL) and water (4 mL), and then stirred at 95 °C for 30 min under $N_2$. After cooling to room temperature, water (20 mL) was added. Product was extracted with ethyl acetate (20 mL) three times. The organic layer was washed with brine (10 mL), and dried over anhydrous $Na_2SO_4$. After filtration, solvents were removed under reduced pressure to yield a crude solid (1.1 g). The crude solid was washed with diisopropyl ether, and yellow solid **3** was obtained (0.52 g, 2.0 mmol, Yield 61%). Compound **3** was used in the next step without further purification.

Synthesis of **4**. *N*-Bromosuccinimide (0.35 g, 2.0 mmol) was added to compound **3** (0.52 g, 2.0 mmol) dissolved in *N,N*-dimethylformamide (33 mL) at 0 °C. The solution was allowed to warm to room temperature, and stirred for 1 hour. Precipitated solid was collected by filtration, and washed with diisopropyl ether. Thus, compound **4** was obtained as a yellow solid (0.29 g, 0.85 mmol, Yield 43%). $^{1}$H-NMR (400 MHz, DMSO-$d_6$): $\delta$ = 9.47 (m, 1H), 8.99 (m, 1H), 8.55 (s, 1H), 8.38 (s, 1H), 7.79 (d, $J$ = 8.0 Hz, 1H), 7.67 (t, $J$ = 7.7 Hz, 1H), 7.46 (d, $J$ = 8.0 Hz, 1H), 7.42 (t, $J$ = 7.7 Hz, 1H) ppm; $^{13}$C NMR (101 MHz, DMSO-$d_6$): $\delta$ = 160.0, 153.7, 151.4, 146.1, 144.4, 142.1, 135.9, 132.9, 129.4, 125.4, 120.9, 119.7, 117.5, 116.6, 84.0 ppm; FT-IR(ATR): ν = 3128, 3060, 1697, 1679, 1606 cm$^{-1}$; HRMS (ESI-MS, methanol with 0.1% formic acid); calculated for [$C_{15}H_9N_3O_2{}^{79}Br$] = 341.9873, found: = 341.9884.

Synthesis of **PC**. Compound **4** (0.29 g, 0.85 mmol), 3-pyridylboronic acid (**5**) (0.40 g, 3.3 mmol), tetrakis(triphenylphosphine)palladium(0) (0.14 g, 0.12 mmol), and $Cs_2CO_3$ (0.57 g, 1.7 mmol) were dispersed in a mixture of 1,4-dioxane (12 mL) and water (1.5 mL), and then stirred at 95 °C for 3.5 hour under $N_2$. After cooling, precipitated solid was collected by filtration, and then washed with ethyl acetate and methanol. The crude product was purified by medium pressure column chromatography (neutral silica gel 10 g, $CH_2Cl_2$/Methanol = 99/1 ~ 0/100), and the solid product was washed with methanol. Thus, compound **PC** was obtained as a yellow solid (0.14 g, 0.41 mmol, Yield 49%). $^{1}$H-NMR (400 MHz, DMSO-$d_6$): $\delta$ = 9.52 (d, $J$ = 1.8 Hz, 1H), 9.32 (s, 1H), 9.06 (d, $J$ = 2.3 Hz, 1H), 8.84 (s, 1H), 8.57 (s, 1H), 8.48-8.46 (m, 2H), 7.80 (dd, $J$ = 7.8, 1.4 Hz, 1H), 7.70-7.66 (m, 1H), 7.48-7.41 (m, 3H) ppm; $^{13}$C NMR (101 MHz, DMSO-$d_6$): $\delta$ = 159.1, 152.8, 150.1, 146.6, 146.5, 143.3, 143.1, 140.9, 134.9, 132.3, 131.9, 128.4, 127.3, 124.4, 123.2, 120.1, 118.8, 116.2, 115.6, 106.2 ppm; FT-IR(ATR): ν = 3037, 1714, 1625, 1606 cm$^{-1}$; HRMS (ESI-MS, methanol with 0.1% formic acid); calculated for [$C_{20}H_{13}N_4O_2$] = 341.1033, found: = 341.1043.

**Table S3.** Summary of HRMS results of **PC** and its precursor **4**.

| Compound | Molecular Formula | Observed Ion | Theoretical Mass | Observed Mass | Error (ppm) |
|---|---|---|---|---|---|
| **4** | $C_{15}H_9N_3O_2{}^{79}Br$ | $[M+H]^+$ | 341.9873 | 341.9884 | 3.24 |
| **PC** | $C_{20}H_{13}N_4O_2$ | $[M+H]^+$ | 341.1033 | 341.1043 | 2.86 |

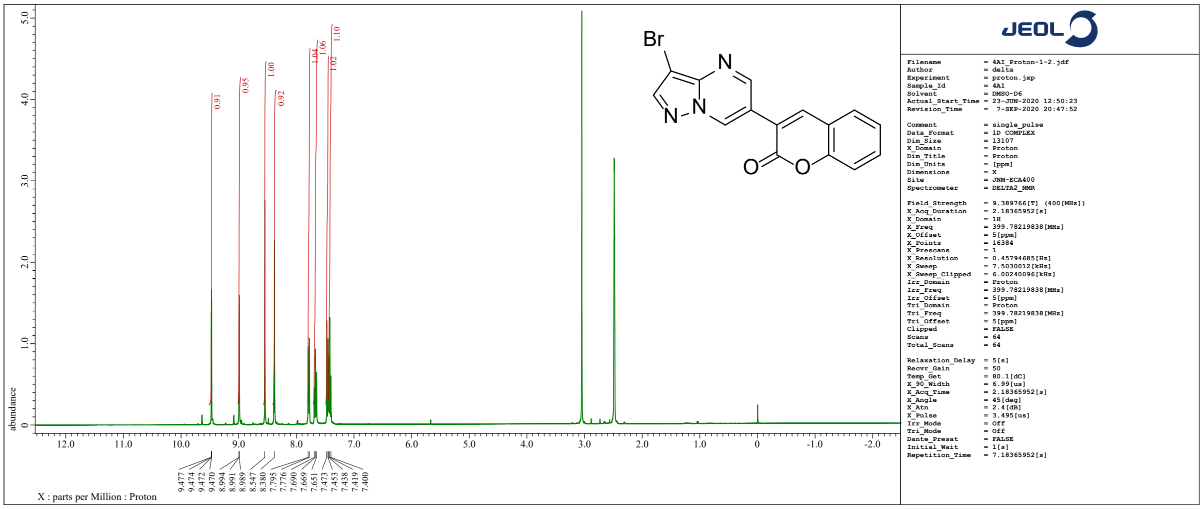

**Figure S5. $^{1}$H NMR spectrum of 4 in DMSO-$d_6$.**

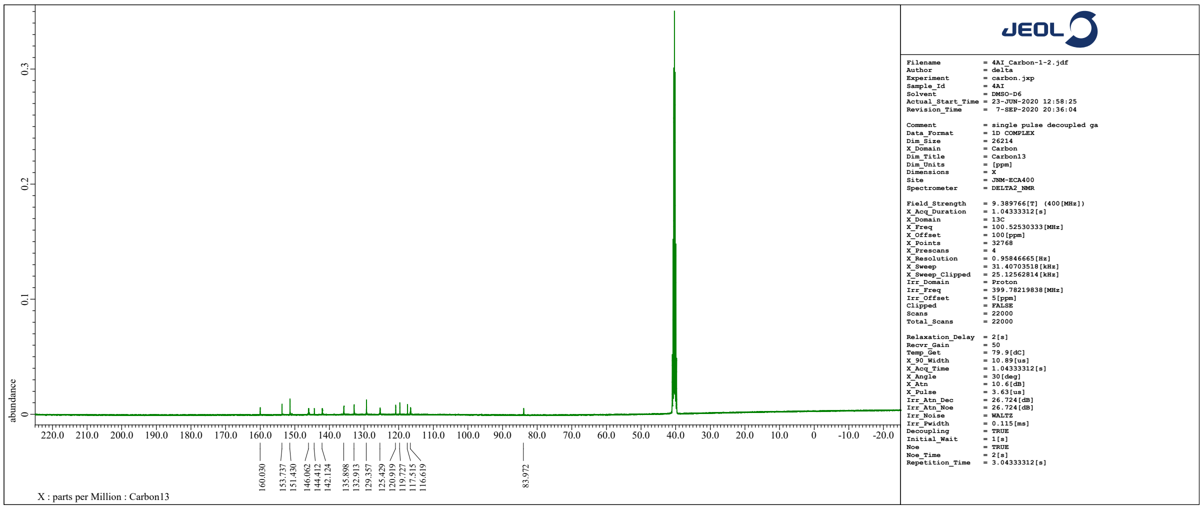

**Figure S6. $^{13}$C NMR spectrum of 4 in DMSO-$d_6$.**

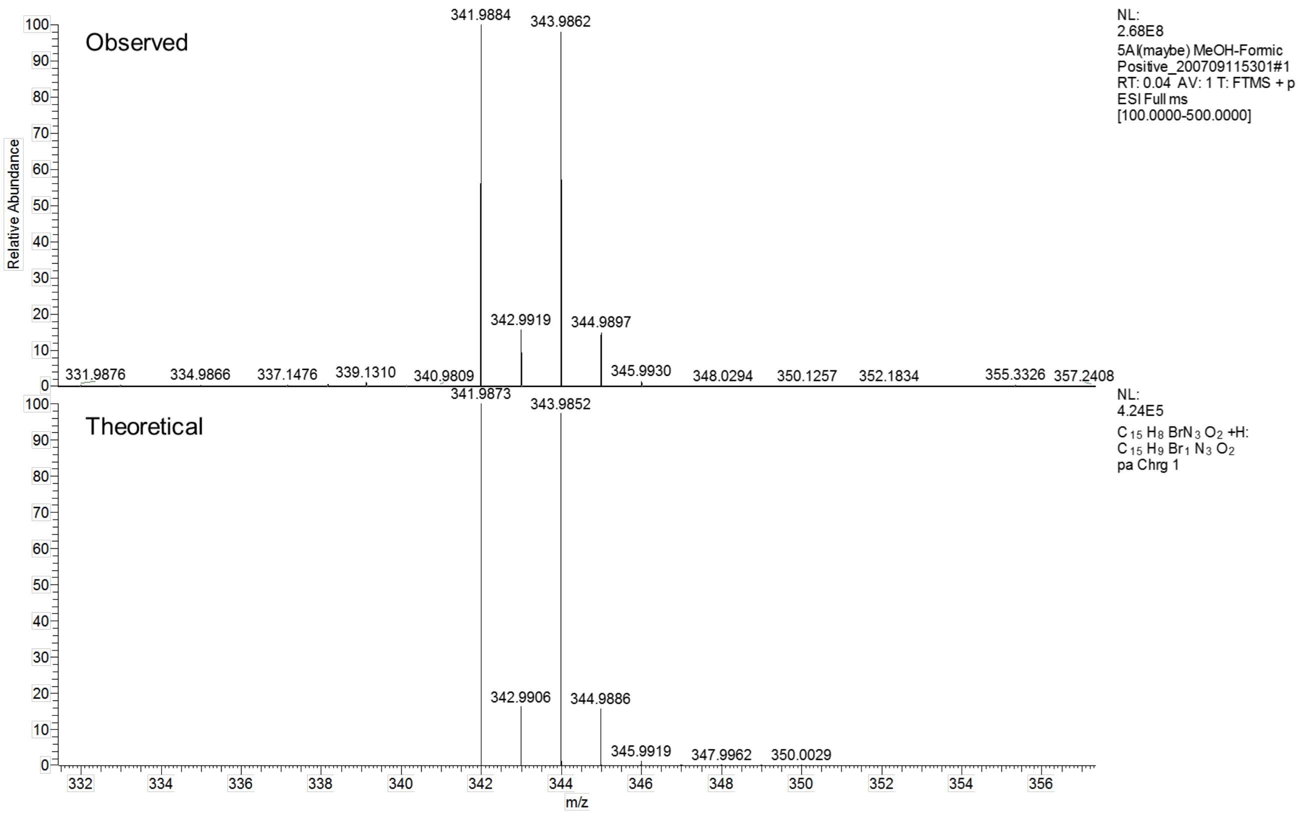


**Figure S7. HRMS analysis of compound 4.**

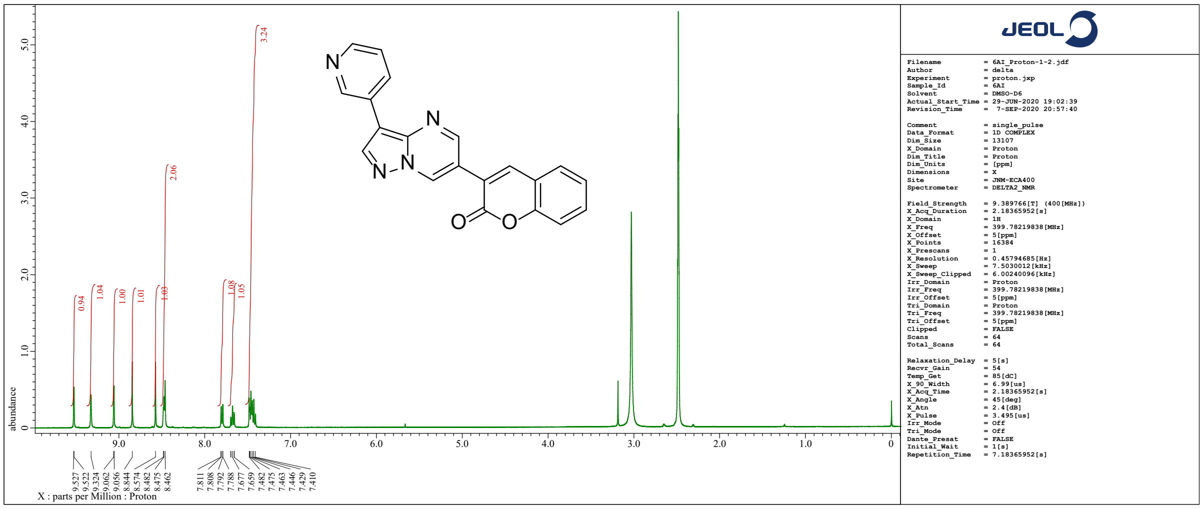

**Figure S8. $^{1}H$ NMR spectrum of PC in DMSO-$d_6$.**

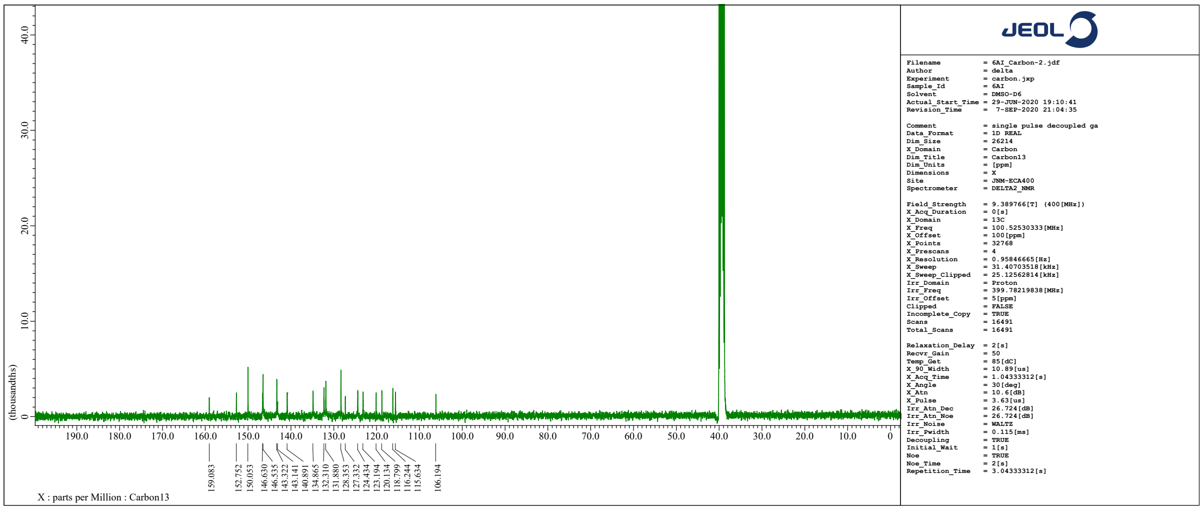

**Figure S9. $^{13}C$ NMR spectrum of PC in DMSO-$d_6$.**

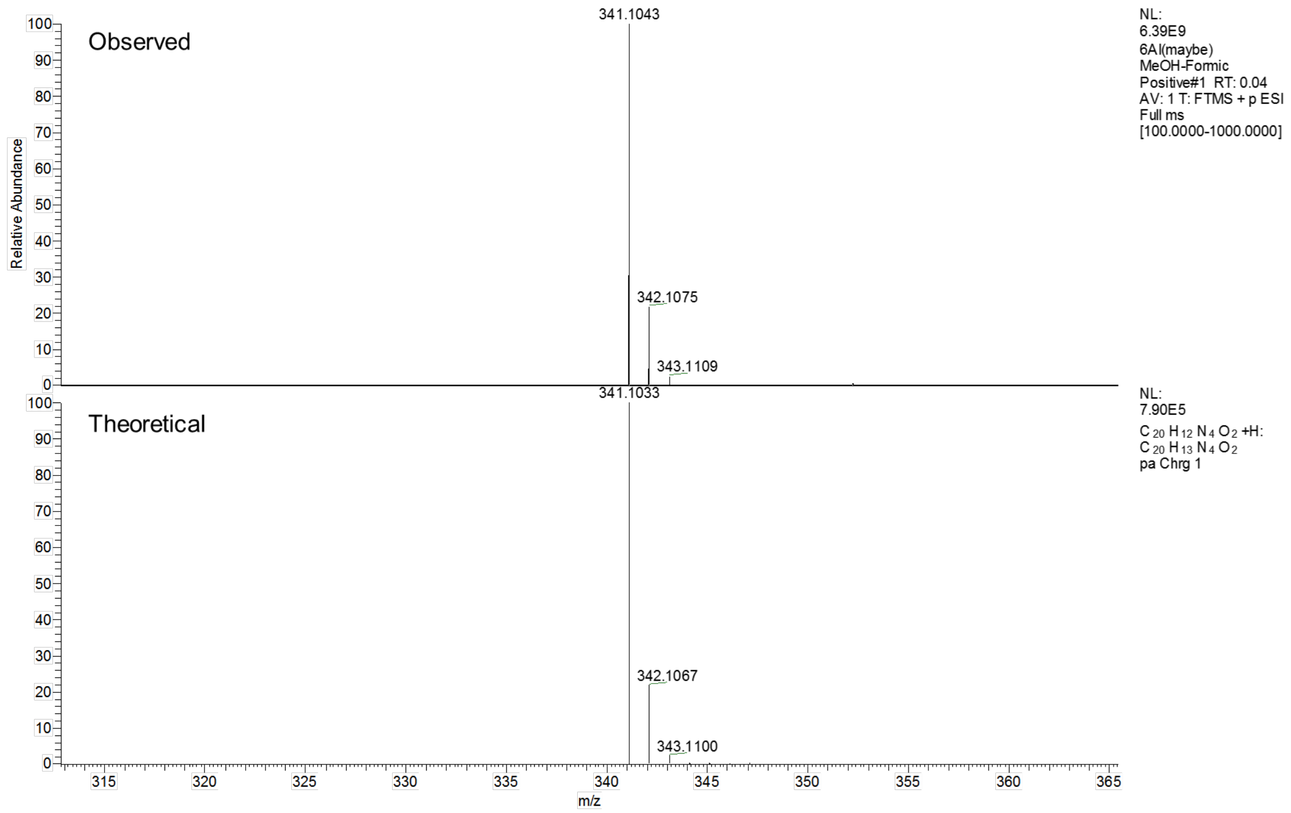


**Figure S10. HRMS analysis of compound PC**.

## 4.4. Concentration dependence of photoluminescence spectra of PC

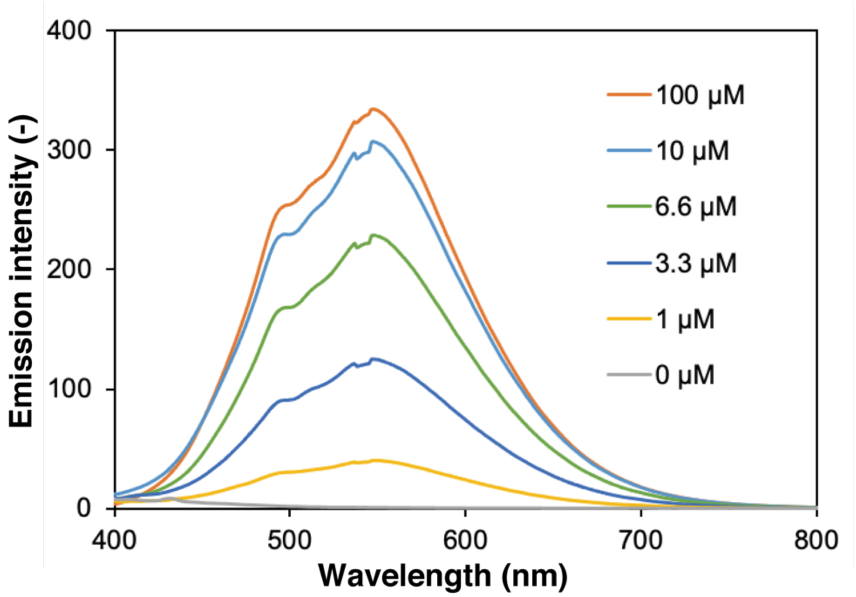


**Figure S11**. **Photoluminescence (PL) spectra of several concentration of PC in DCM solvent.** The wavelength of excitation light is 350 nm.

### 4.5. Photoluminescence (PL) spectra of PC under $N_2$

PL spectra under air and $N_2$ (after $N_2$ bubble for 20 min) are measured at room temperature. Since there is no difference between them, phosphorescence should not be involved in PL of **PC**.

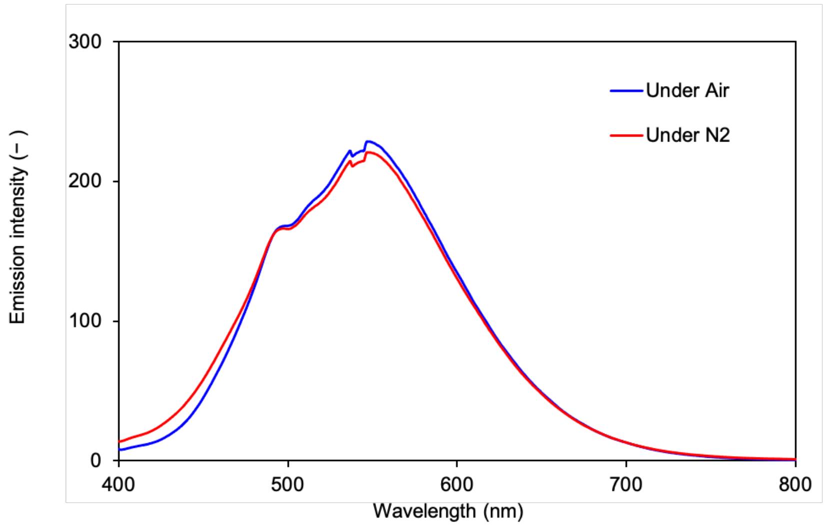


**Figure S12**. **PL spectra of PC under air and $N_2$.**

## 5. Experimental Details of known molecules (A-G)

### 5.1. Materials

The materials of **A**-**G** (Table S2) were obtained from Tokyo Chemical Industry Co., Ltd. through custom synthesis. Spectroscopic grade solvents (DCM or THF) were obtained from Fujifilm Wako Pure Chemical Corporation.

### 5.2 Photophysical properties of A-G

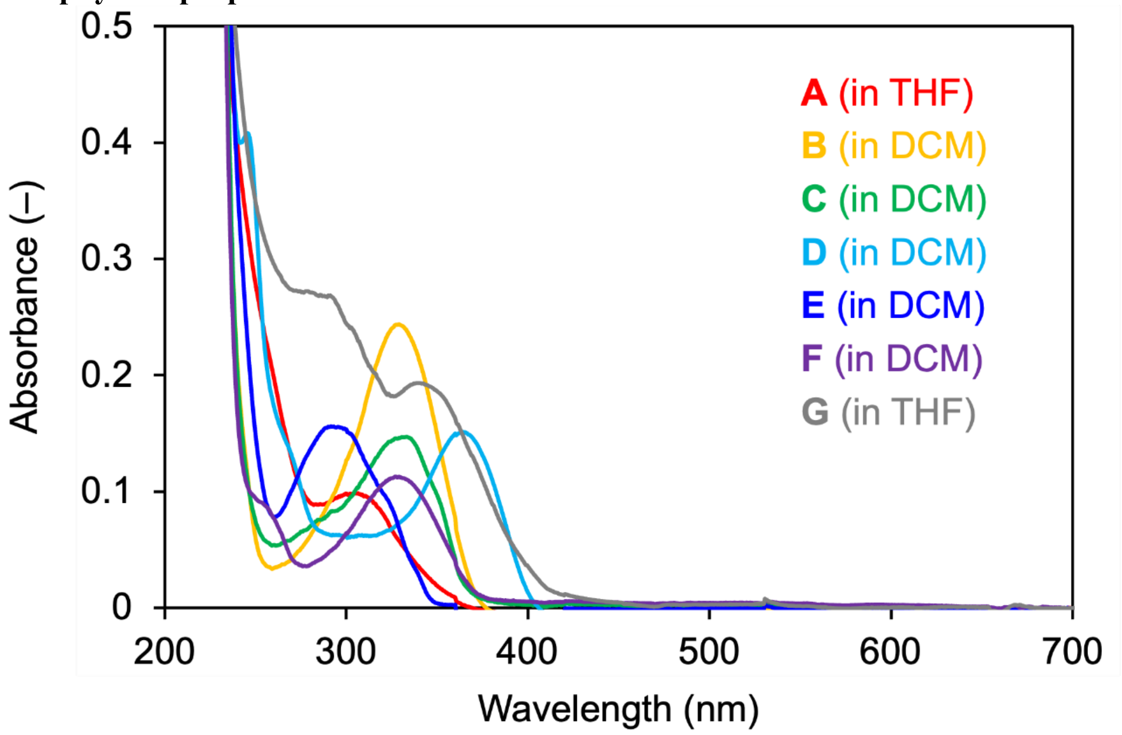


**Figure S13. UV-vis absorption spectra of compounds A–G in DCM or THF (10 μM, 1 cm optical length).**

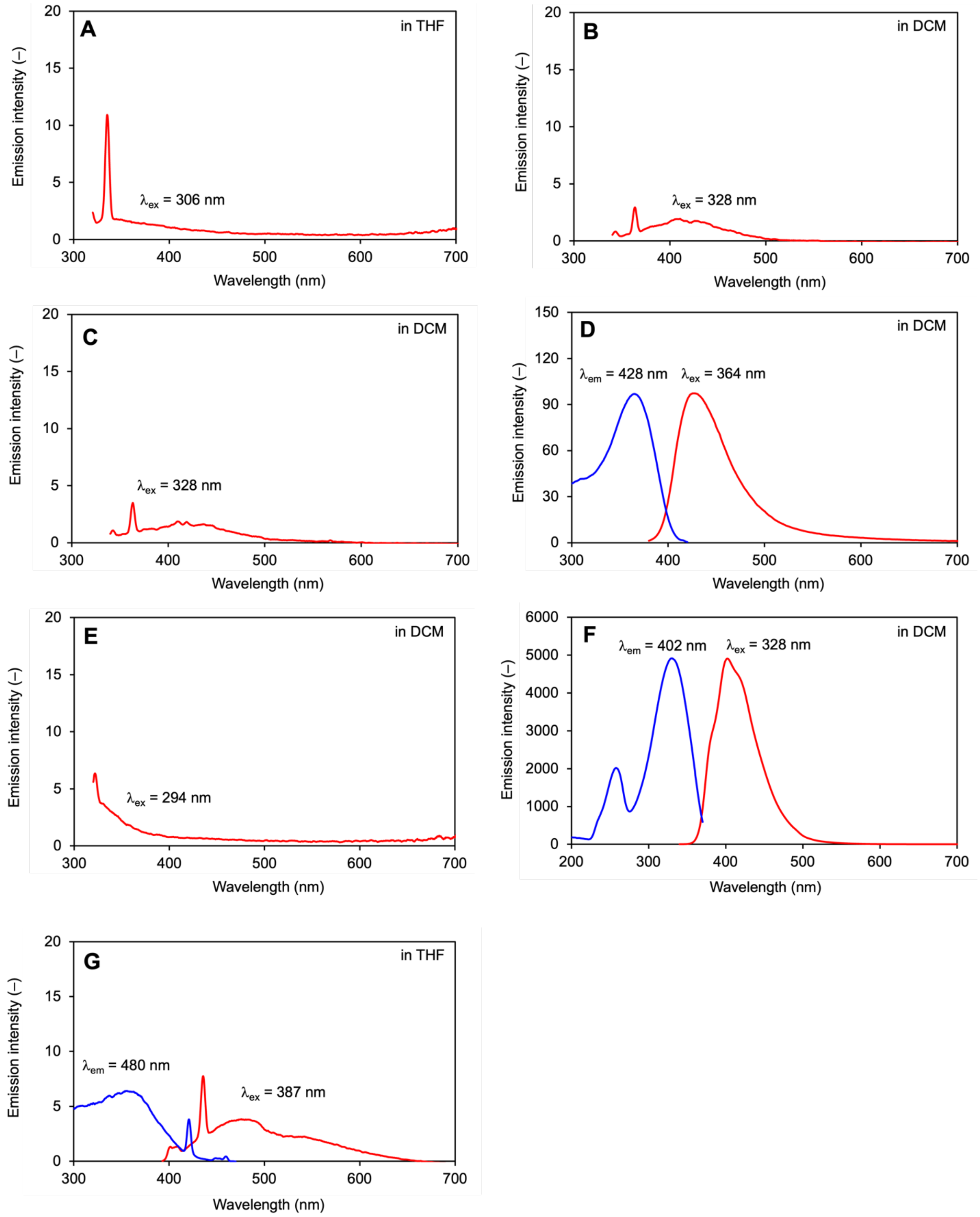


**Figure S14**. **PL spectra of compounds A–G in DCM or THF (10 µM, 1 cm optical length).** Note that emission from compounds **A**, **B**, **C**, **E**, and **G** are negligibly small.

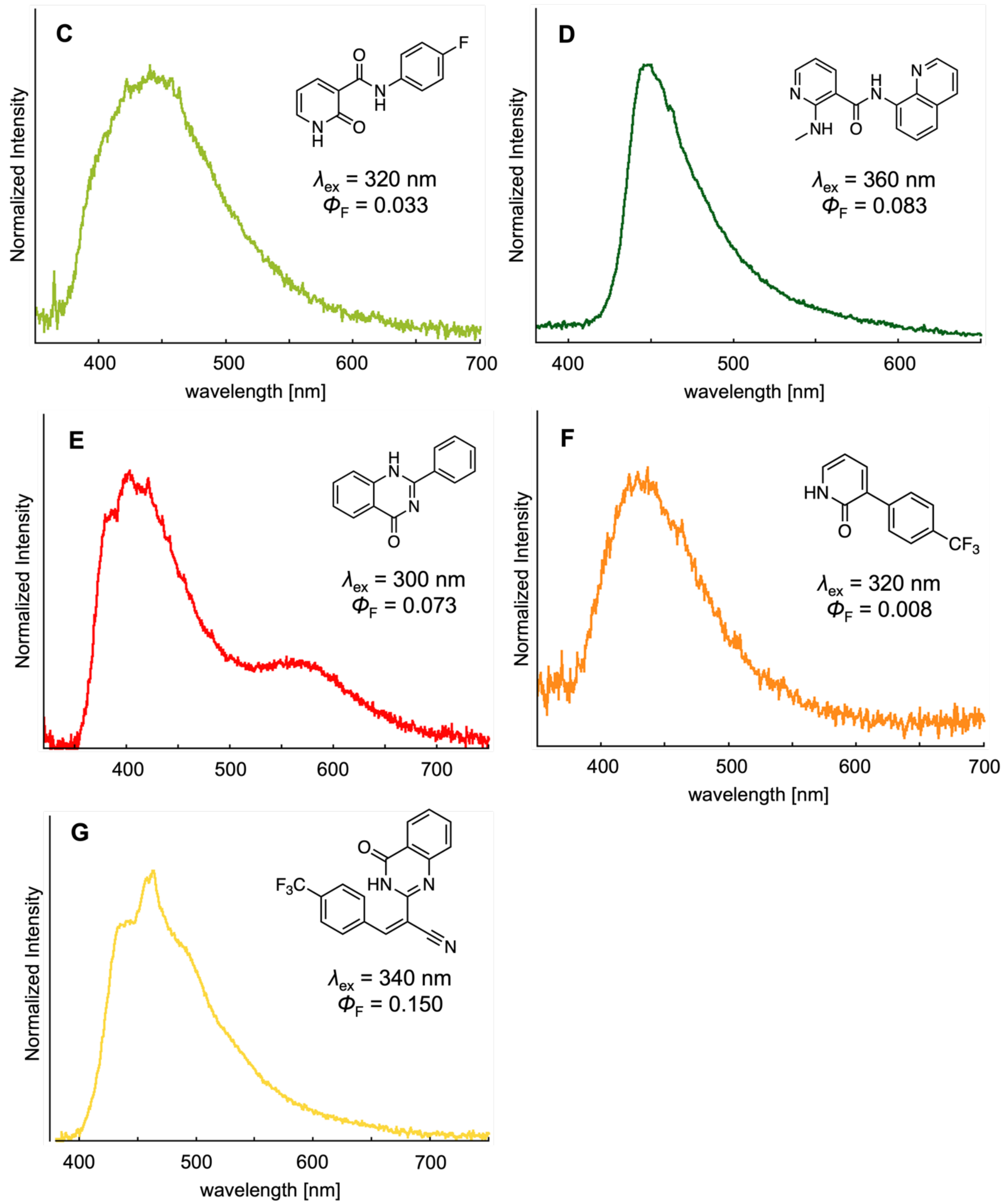


**Figure S15. Solid state PL spectra of C-G.** Solid state PL spectra of **A** and **B** were not detected.

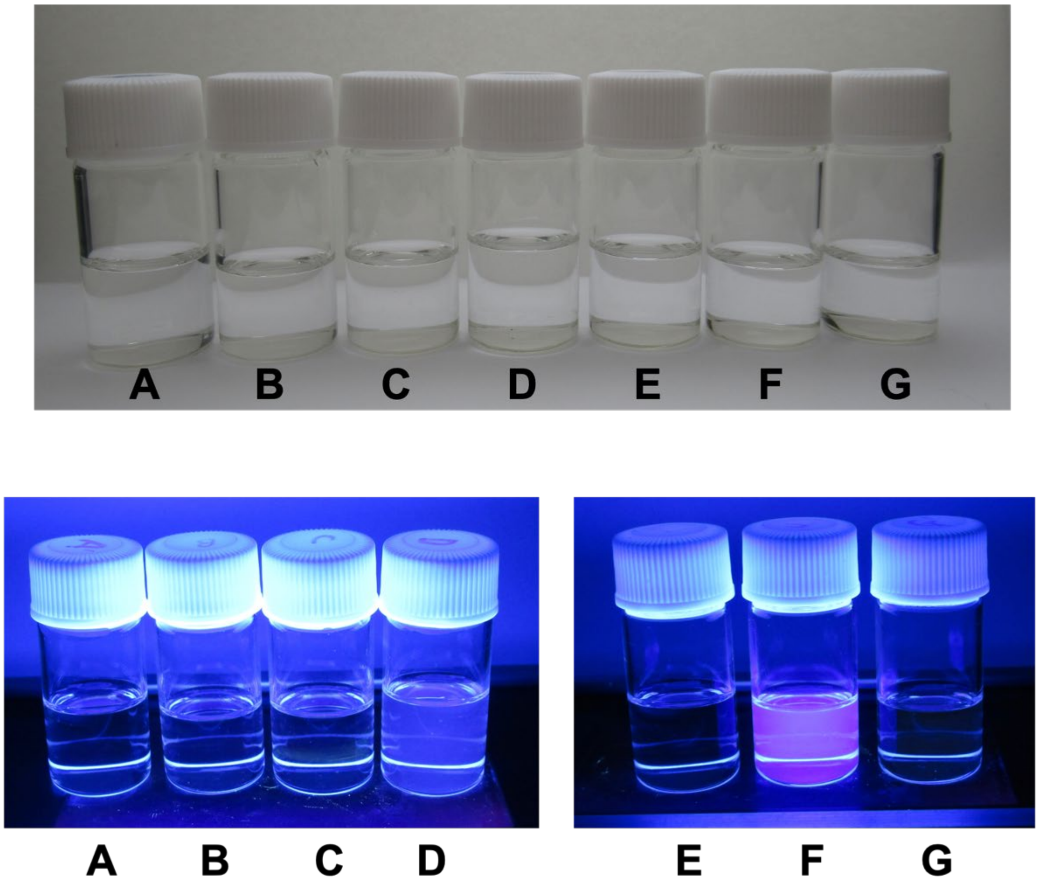


**Figure S16. Photo images of compounds A–G. A** and **G** are in THF solvent, **B**–**F** are in DCM with 10 μM under room light (up) and UV light at 365 nm (bottom).

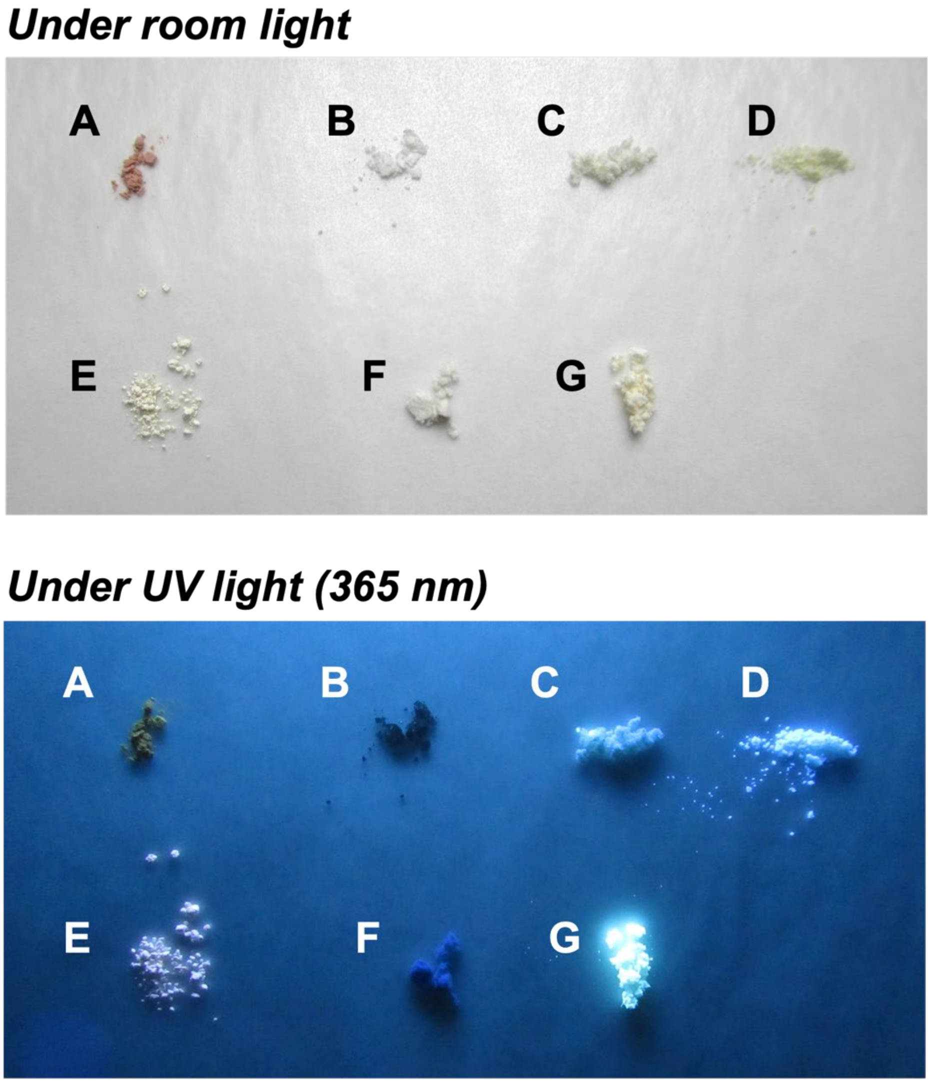


**Figure S17. Photo images of compounds A–G in solid state.** Upper one is under room light and bottom is UV light at 365 nm.

### 5.3 Characterization of A-G

ATR-FTIR spectra were obtained using a Thermo-Nicolet 760X FTIR spectrophotometer equipped with a SMART-iTX ATR accessory. $^{1}$H-NMR spectra were obtained using a JEOL JNM-ECA400 or JNM-ECZ400S spectrometer operating at 400 MHz and using tetramethylsilane (TMS) as an internal standard. Proton decoupled $^{13}$C-NMR spectra were obtained using a JEOL JNM-ECA400 or JNM-ECZ400S spectrometer operating at 101 MHz and using TMS as an internal standard. $^{19}$F-NMR spectra were obtained using a JEOL JNM-ECZ400S spectrometer operating at 376 MHz. Data was processed using Delta version 5.0.5.1. $^{1}$H NMR chemical shifts ($\delta$) are reported in ppm relative to TMS in DMSO-$d_6$ ($\delta$ = 0.00). $^{13}$C NMR chemical shifts ($\delta$) are reported in ppm relative to the solvent reported. Coupling constants ($J$) are expressed in Hertz (Hz), shift multiplicities are reported as singlet (s), doublet (d), triplet (t), quartet (q), double doublet (dd), multiplet (m) and broad singlet (bs). High resolution ESI-MS mass spectra were

measured using a Thermo Scientific Q-Exactive Plus instrument in methanol with 0.1% formic acid or methanol with 10% tetrahydrofuran.

**Table S4.** Summary of HRMS results.

| Compound | Molecular Formula | Observed Ion | Theoretical Mass | Observed Mass | Error (ppm) |
|---|---|---|---|---|---|
| **A** | $C_{10}H_8N_6O_2$ | $[M–H]^–$ | 243.0625 | 243.0633 | 3.09 |
| **B** | $C_{15}H_{16}N_2O_2$ | $[M+H]^+$ | 257.1285 | 257.1280 | –1.69 |
| **C** | $C_{12}H_9N_2O_2F$ | $[M–H]^–$ | 231.0564 | 231.0570 | 0.61 |
| **D** | $C_{16}H_{14}N_4O$ | $[M+H]^+$ | 279.1240 | 279.1237 | –1.35 |
| **E** | $C_{14}H_{10}N_2O$ | $[M–H]^–$ | 221.0709 | 221.0715 | 2.54 |
| **F** | $C_{12}H_8NOF_3$ | $[M–H]^–$ | 238.0474 | 238.0481 | –3.00 |
| **G** | $C_{18}H_{10}N_3OF_3$ | $[M–H]^–$ | 340.0692 | 340.0700 | 2.40 |

4-amino-*N*-(1*H*-indazol-5-yl)-1,2,5-oxadiazole-3-carboxamide (**A**)

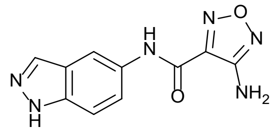

$^1$H-NMR (400 MHz, DMSO-*d*6): $\delta$ = 13.10 (s, 1H), 11.01 (s, 1H), 8.24 (s, 1H), 8.09 (s, 1H), 7.66 (dd, *J* = 8.9, 1.8 Hz, 1H), 7.55 (d, *J* = 8.9 Hz, 1H), 6.46 (s, 2H) ppm; $^{13}$C NMR (101 MHz, DMSO-*d*6): $\delta$ = 157.0, 156.7, 141.7, 137.9, 134.2, 131.1, 123.1, 121.8, 112.4, 110.8 ppm; FT-IR(ATR): ν = 3473, 3395, 3329, 1689, 1619, 1595, 1562, 1512 cm$^{-1}$; HRMS (ESI-MS, methanol with 10% THF); calculated for [$C_{10}H_7N_6O_2$] = 243.0625, found: = 243.0633.

3-(4-methoxyphenyl)-2-(pyrrolidine-1-carbonyl)acrylonitrile (**B**)

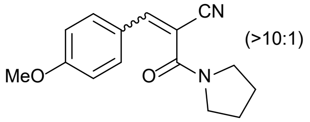

$^1$H-NMR (400 MHz, DMSO-*d*6): $\delta$ = 7.97 (d, *J* = 8.7 Hz, 2H), 7.88 (s, 1H), 7.12 (d, *J* = 8.7 Hz, 2H), 3.64 (t, *J* = 6.3 Hz, 2H), 3.43 (t, *J* = 6.3 Hz, 2H), 1.91-1.84 (m, 4H) ppm; $^{13}$C NMR (101 MHz, DMSO-*d*6): $\delta$ = 162.9, 161.9, 151.0, 132.6, 125.3, 117.4, 115.2, 103.7, 56.1, 48.9, 47.4, 26.6, 24.3 ppm; FT-IR(ATR): ν = 2198, 1630, 1585, 1511 cm$^{-1}$; HRMS (ESI-MS, methanol with 10% THF); calculated for [$C_{15}H_{17}N_2O_2$] = 257.1285, found: = 257.1280. Note that this product is a mixture of E/Z forms.

*N*-(4-fluorophenyl)-2-oxo-1,2-dihydropyridine-3-carboxamide (**C**)

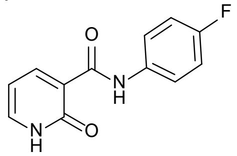

$^1$H-NMR (400 MHz, DMSO-*d*6): $\delta$ = 12.77 (s, 1H), 12.20 (s, 1H), 8.48 (dd, *J* = 7.1, 2.1 Hz, 1H), 7.83 (dd, *J* = 6.4, 2.3 Hz, 1H), 7.73 (q, *J* = 4.7 Hz, 2H), 7.21 (t, *J* = 8.9 Hz, 2H), 6.59 (t, *J* = 6.6 Hz, 1H) ppm; $^{13}$C NMR (101 MHz, DMSO-*d*6): $\delta$ = 163.1, 162.0, 160.0, 157.6, 145.1, 140.7, 135.3,

122.0, 121.9, 120.4, 116.2, 116.0, 107.4 ppm; $^{19}$F-NMR (376 MHz, DMSO-$d_6$) δ = -118.6 ppm; FT-IR(ATR): ν = 2500-3100 (broad), 3064, 1677, 1642, 1607, 1545, 1508 cm$^{-1}$; HRMS (ESI-MS, methanol with 10% THF); calculated for [$C_{12}H_9N_2O_2F$] = 231.0564, found: = 231.0570.

2-(methylamino)-*N*-(quinolin-8-yl)nicotinamide (**D**)

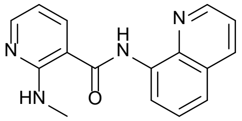

$^1$H-NMR (400 MHz, DMSO-$d_6$): $\delta$ = 10.58 (s, 1H), 8.97 (d, $J$ = 2.8 Hz, 1H), 8.63 (d, $J$ = 7.8 Hz, 1H), 8.46 (d, $J$ = 8.3 Hz, 1H), 8.31 (d, $J$ = 4.1 Hz, 1H), 8.12-8.06 (m, 2H), 7.76-7.63 (m, 3H), 6.73 (dd, $J$ = 7.3, 4.6 Hz, 1H), 2.98 (s, 3H) ppm; $^{13}$C NMR (101 MHz, DMSO-$d_6$): $\delta$ = 166.4, 158.5, 152.6, 149.7, 139.0, 137.3, 136.6, 134.5, 128.4, 127.5, 123.0, 122.8, 117.5, 111.3, 110.7, 28.4 ppm; FT-IR(ATR): ν = 3351, 3317, 1647, 1591, 1576, 1517 cm$^{-1}$; HRMS (ESI-MS, methanol with 10% THF); calculated for [$C_{16}H_{15}N_4O$] = 279.1240, found: = 279.1237.

2-phenylquinazolin-4(3*H*)-one (**E**)

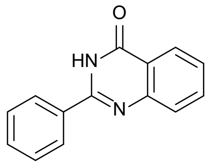

$^1$H-NMR (400 MHz, DMSO-$d_6$): $\delta$ = 12.57 (s, 1H), 8.20-8.16 (m, 3H), 7.85 (t, $J$ = 7.1 Hz, 1H), 7.76 (d, $J$ = 8.3 Hz, 1H), 7.62-7.52 (m, 4H) ppm; $^{13}$C NMR (101 MHz, DMSO-$d_6$): $\delta$ = 162.8, 152.9, 149.2, 135.1, 133.3, 131.9, 129.1, 128.3, 128.0, 127.1, 126.4, 121.5 ppm; FT-IR(ATR): ν = 3061, 1657, 1598, 1571, 1557 cm$^{-1}$; HRMS (ESI-MS, methanol with 10% THF); calculated for [$C_{14}H_9N_2O$] = 221.0709, found: = 221.0715.

3-(4-(trifluoromethyl)phenyl)pyridin-2(1*H*)-one (**F**)

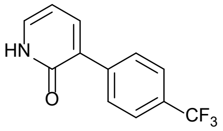

$^1$H-NMR (400 MHz, DMSO-$d_6$): $\delta$ = 11.97 (s, 1H), 7.97 (d, $J$ = 8.5 Hz, 2H), 7.78 (dd, $J$ = 6.9, 2.2 Hz, 1H), 7.74 (d, $J$ = 8.5 Hz, 2H), 7.48 (dd, $J$ = 6.9, 2.2 Hz, 1H), 6.34 (t, $J$ = 6.9 Hz, 1H) ppm; $^{13}$C NMR (101 MHz, DMSO-$d_6$): $\delta$ = 161.5, 141.4, 140.4, 136.5, 129.2, 128.7, 128.1, 127.8, 126.3, 125.3, 125.2, 123.6, 106.0 ppm; $^{19}$F-NMR (376 MHz, DMSO-$d_6$) δ = -60.8 ppm; FT-IR(ATR): ν = 2500-3100 (broad), 1637, 1610, 1557 cm$^{-1}$; HRMS (ESI-MS, methanol with 10% THF); calculated for [$C_{18}H_9N_3OF_3$] = 340.0692, found: = 238.0481.

2-(4-oxo-3,4-dihydroquinazolin-2-yl)-3-(4-(trifluoromethyl)phenyl)acrylonitrile (**G**)

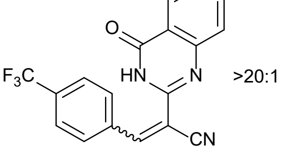

$^{1}$H-NMR (400 MHz, 80 °C, DMSO-$d_6$): $\delta$ = 12.42 (s, 1H), 8.52 (s, 1H), 8.14 (d, $J$ = 7.8 Hz, 1H), 8.10 (d, $J$ = 8.3 Hz, 2H), 7.91 (d, $J$ = 8.3 Hz, 2H), 7.83 (t, $J$ = 7.6 Hz, 1H), 7.72 (d, $J$ = 7.8 Hz, 1H), 7.54 (t, $J$ = 7.6 Hz, 1H) ppm; $^{13}$C NMR (101 MHz, 80 °C, DMSO-$d_6$): $\delta$ = 162.0, 149.1, 148.4, 136.8, 135.3, 132.1, 131.8, 131.0, 128.2, 126.6, 126.5, 126.5, 122.0, 115.6, 110.1, 41.1, 40.9, 40.7, 40.4, 40.2, 40.0, 39.8 ppm; $^{19}$F-NMR (376 MHz, 80 °C, DMSO-$d_6$) δ = -61.6 ppm; FT-IR(ATR): ν = 3065, 2229, 1674, 1618, 1606, 1588, 1563 cm$^{-1}$; HRMS (ESI-MS, methanol with 10% THF); calculated for [$C_{12}H_7NOF_3$] = 238.0474, found: = 340.0700. Note that this product is a mixture of E/Z forms.

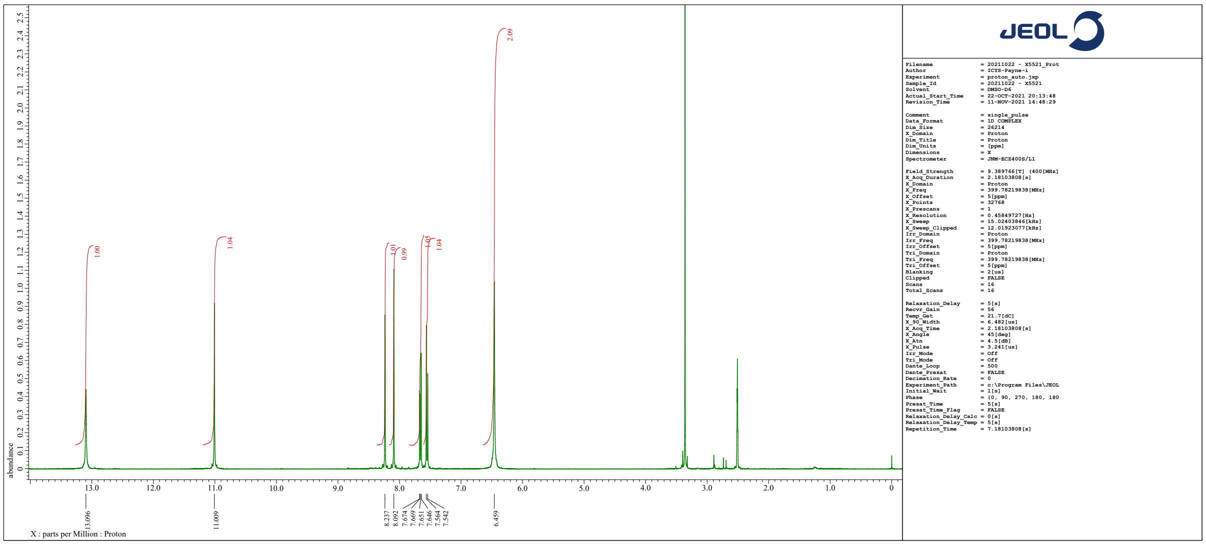


**Figure S18. $^{1}$H NMR spectrum of A in DMSO-$d_6$.**

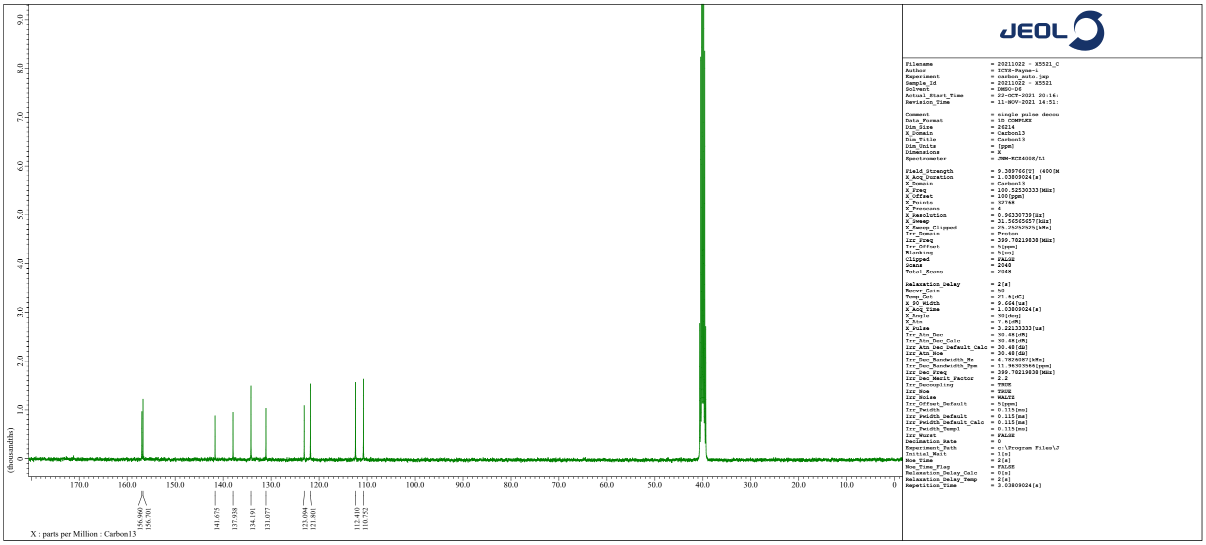


**Figure S19. $^{13}$C NMR spectrum of A in DMSO-$d_6$.**

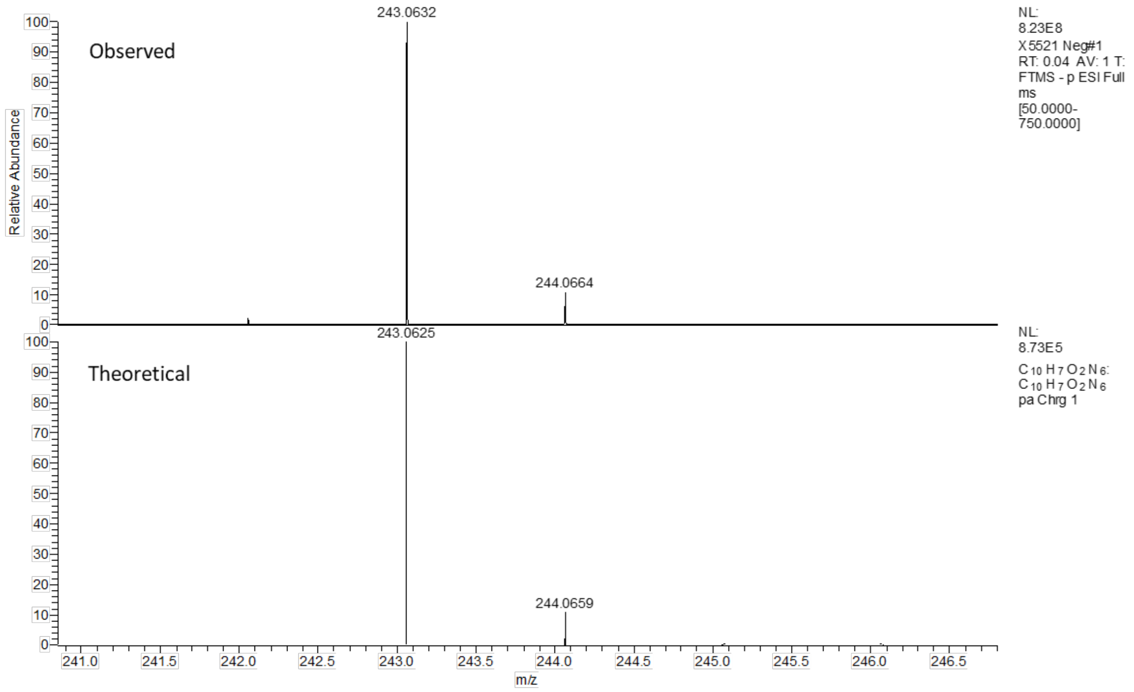


**Figure S20. HRMS analysis of compound A.**

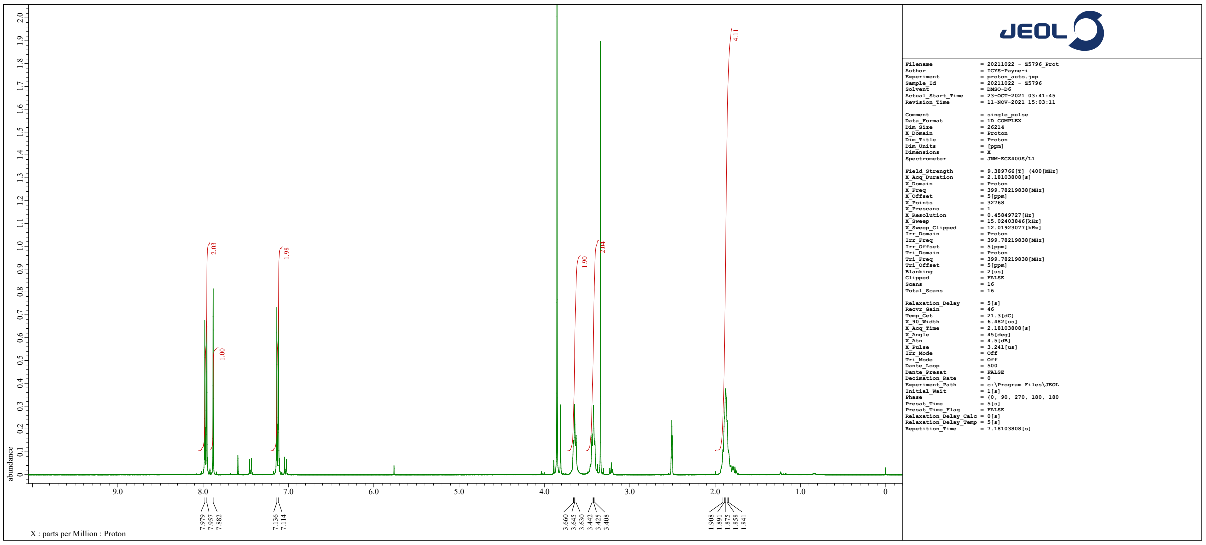


**Figure S21. $^{1}$H NMR spectrum of B in DMSO-$d_6$.**

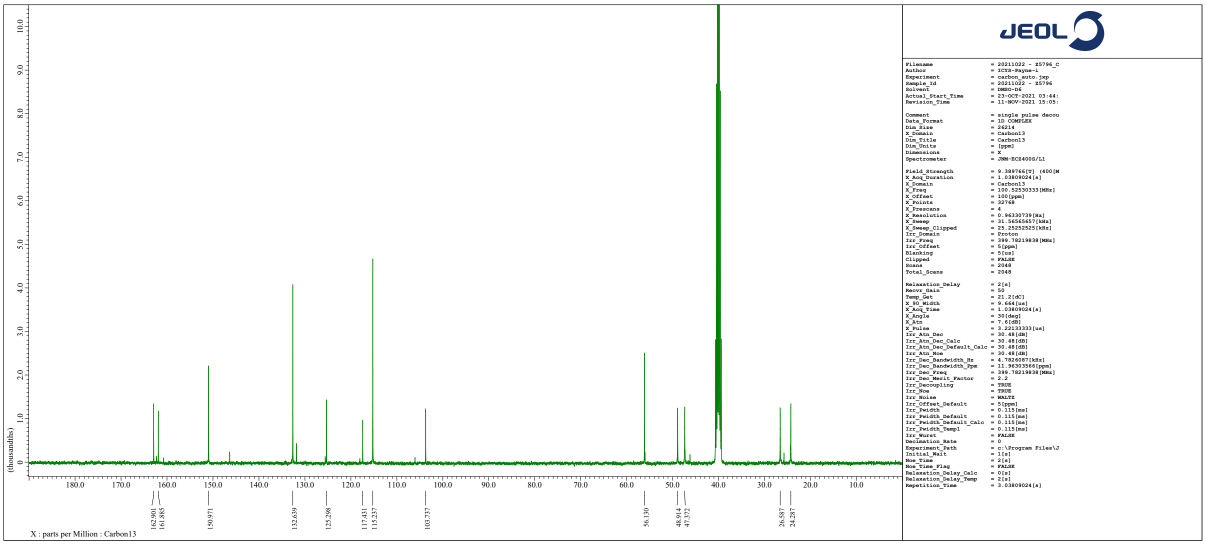


**Figure S22. $^{13}C$ NMR spectrum of B in DMSO-$d_6$.**

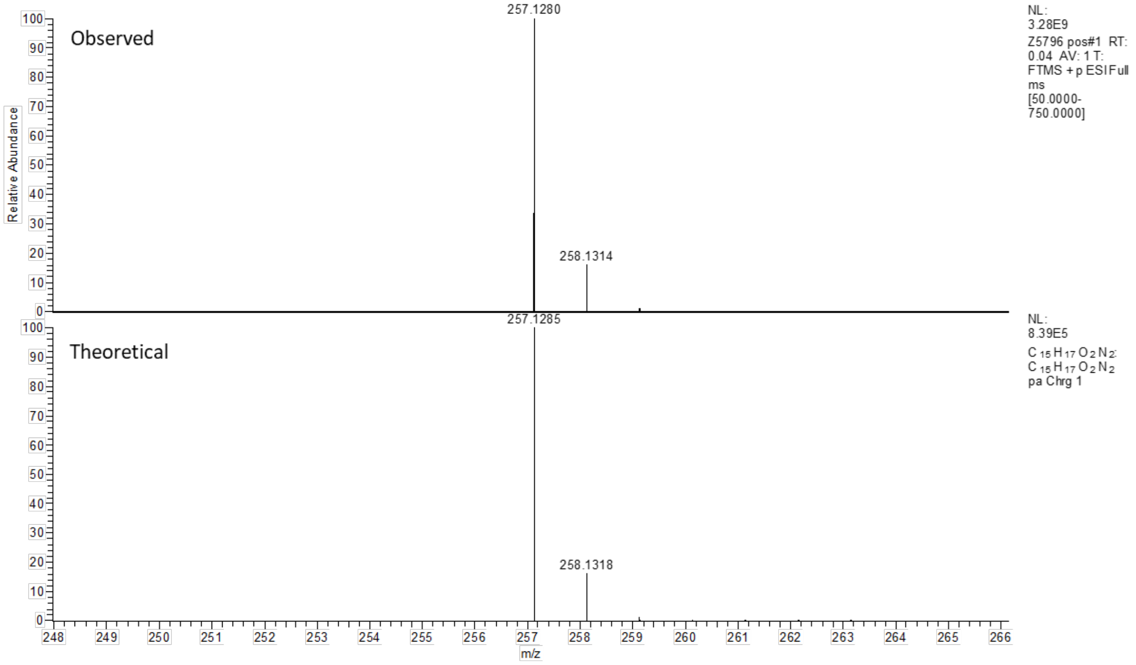


**Figure S23. HRMS analysis of compound B.**

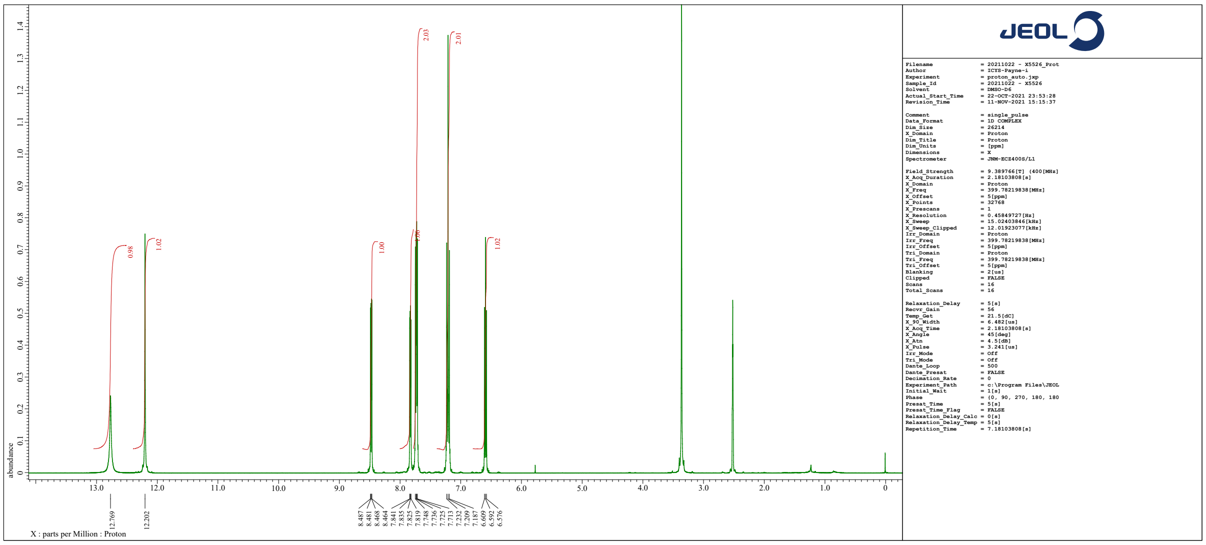


**Figure S24. $^{1}$H NMR spectrum of C in DMSO-$d_6$.**

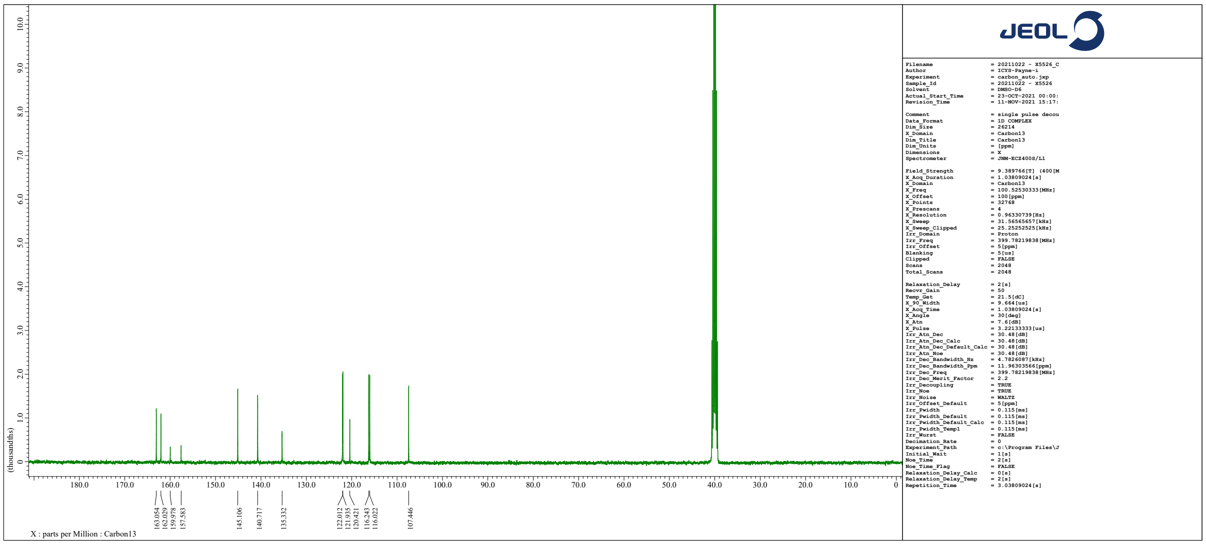


**Figure S25. $^{13}$C NMR spectrum of C in DMSO-$d_6$.**

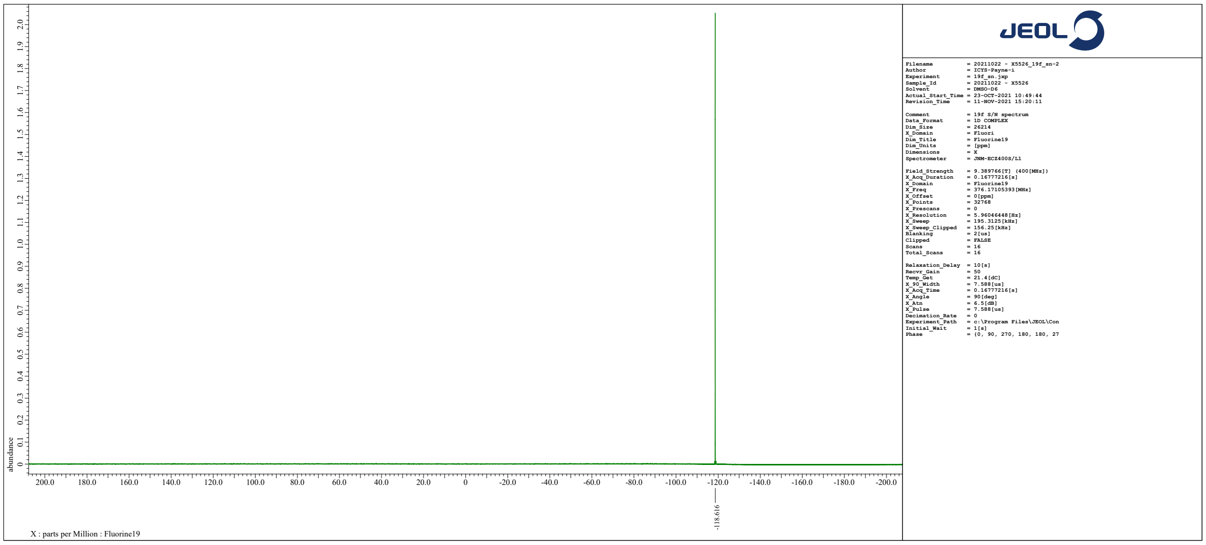


**Figure S26. $^{19}$F NMR spectrum of C in DMSO-$d_6$.**

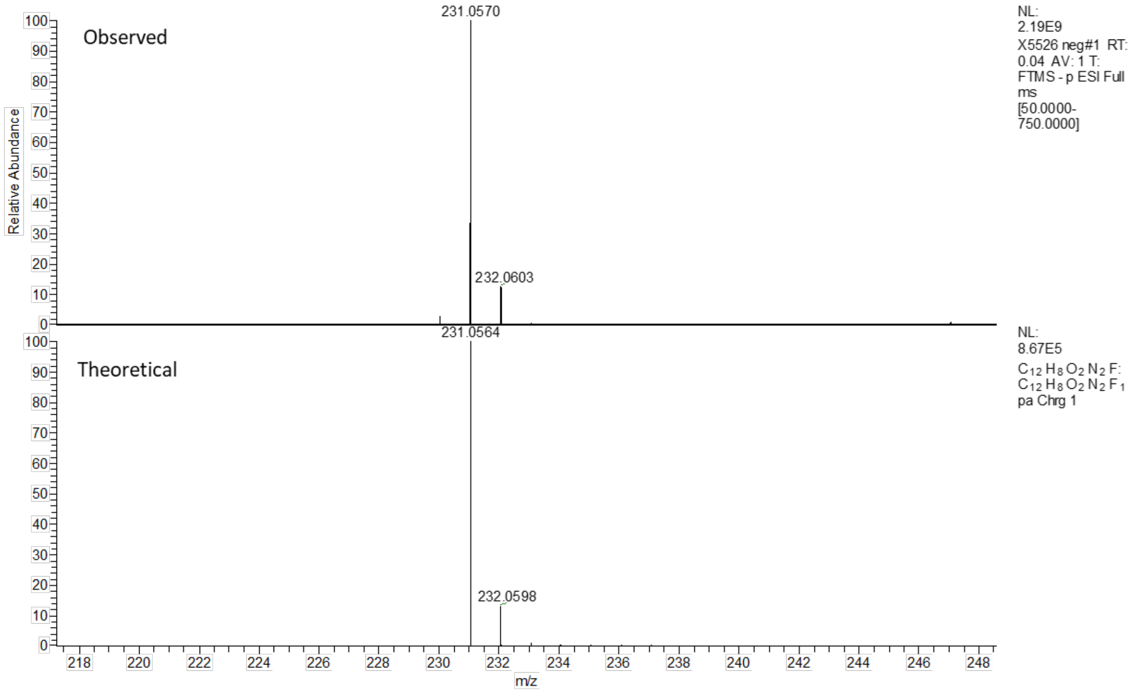


**Figure S27. HRMS analysis of compound C.**

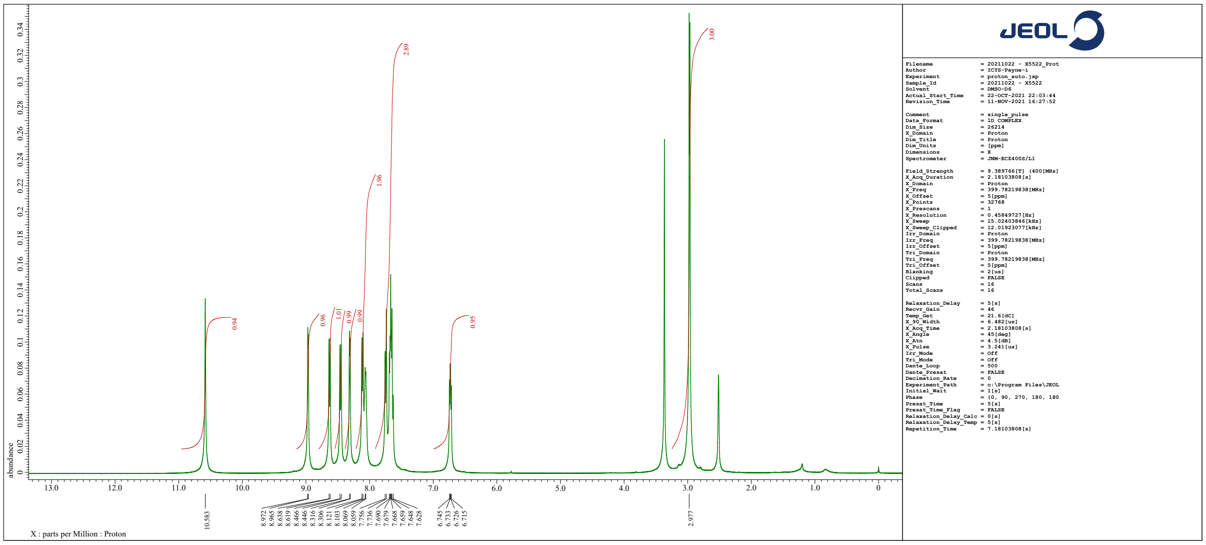


**Figure S28.** $^{1}$H **NMR spectrum of D in DMSO-*d*$_6$.**

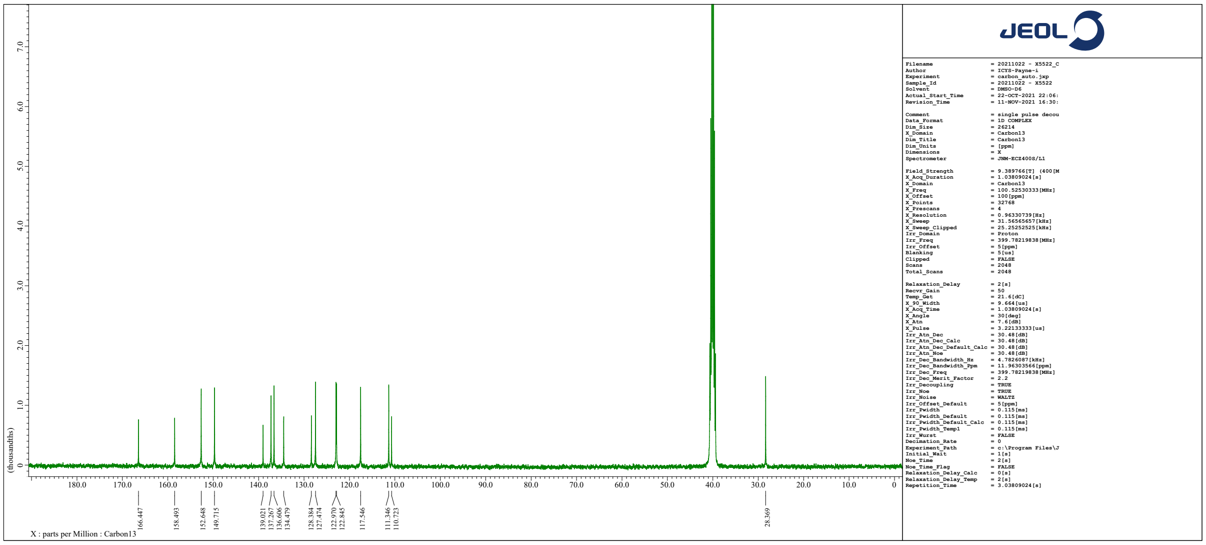


**Figure S29.** $^{13}$C **NMR spectrum of D in DMSO-*d*$_6$.**

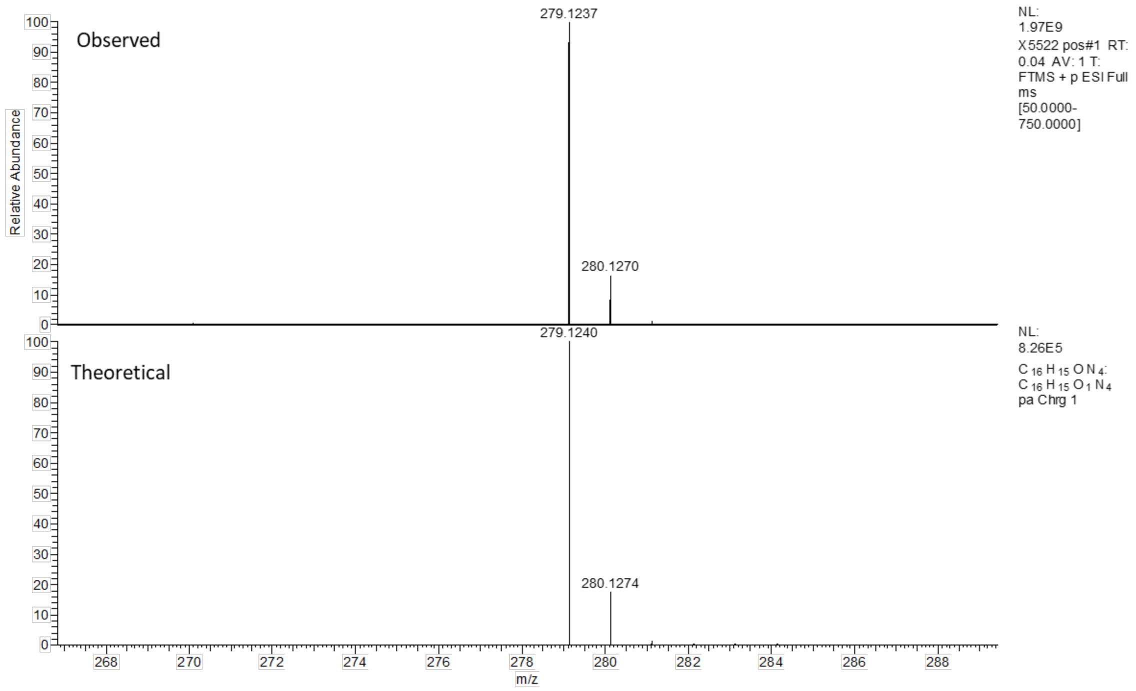


**Figure S30. HRMS analysis of compound D.**

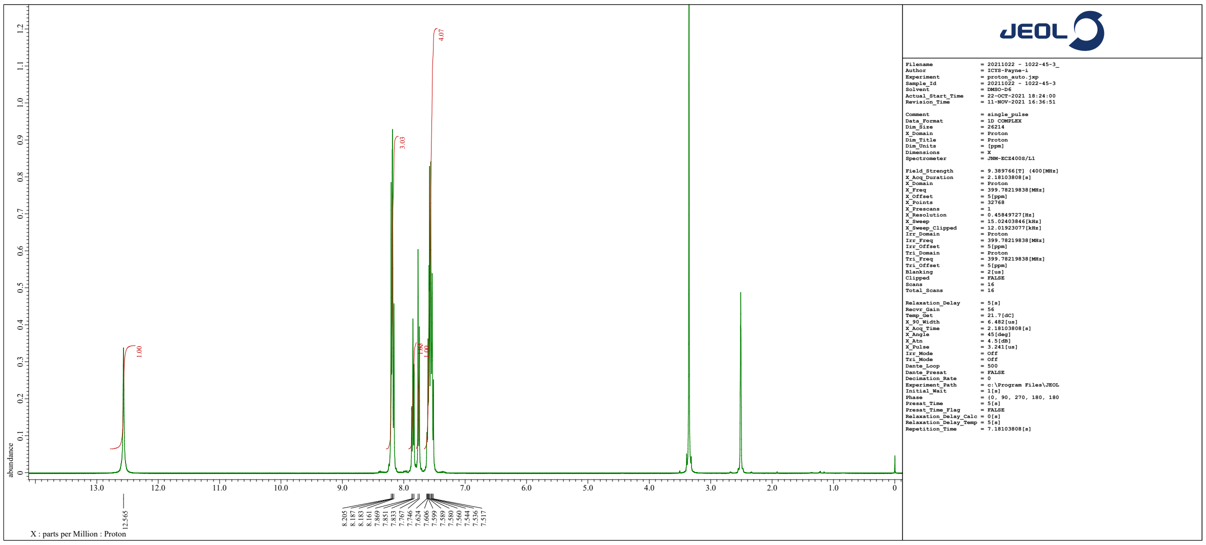


**Figure S31. $^1$H NMR spectrum of E in DMSO-$d_6$.**

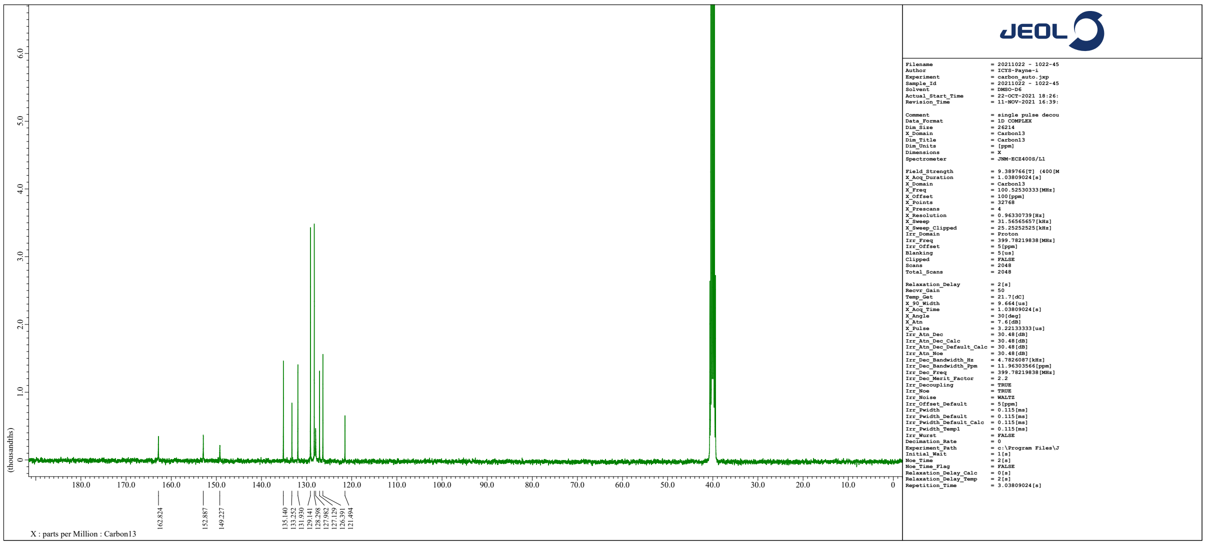

**Figure S32. $^{13}$C NMR spectrum of E in DMSO-$d_6$.**

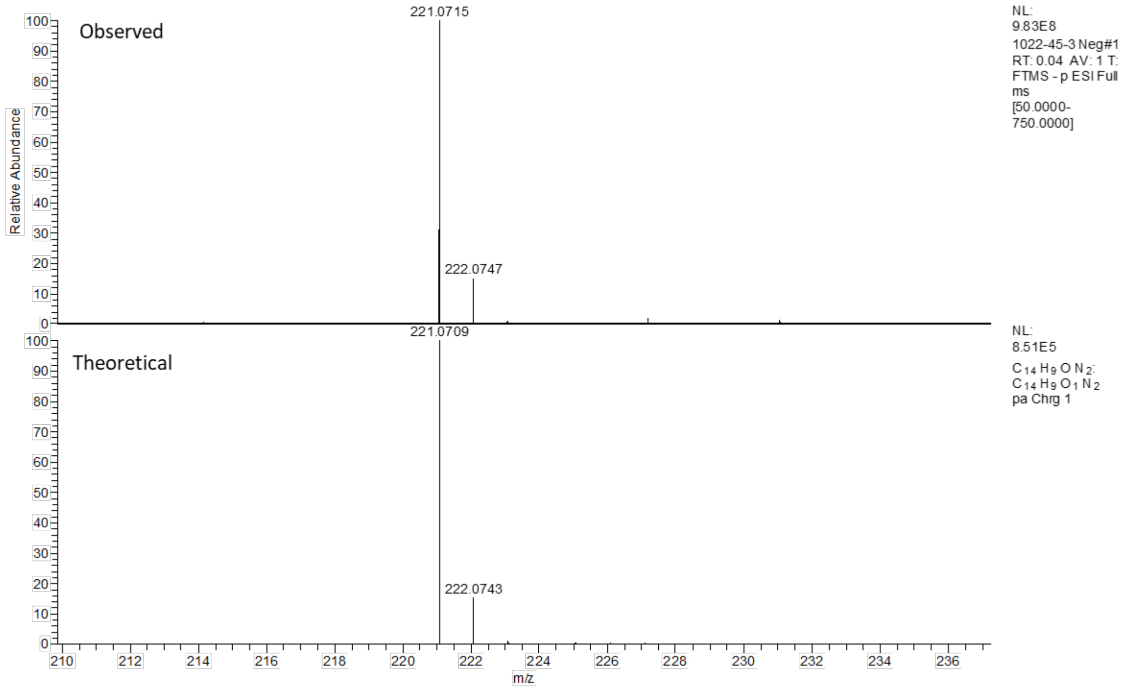


**Figure S33. HRMS analysis of compound E.**

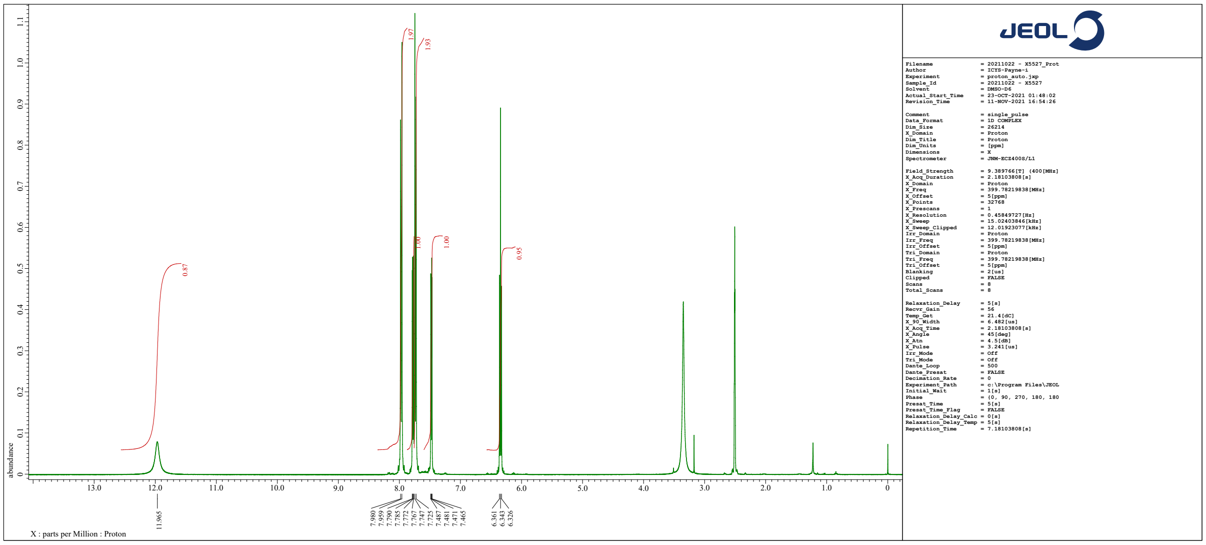


**Figure S34.** $^{1}$**H NMR spectrum of F in DMSO-*d*$_6$.**

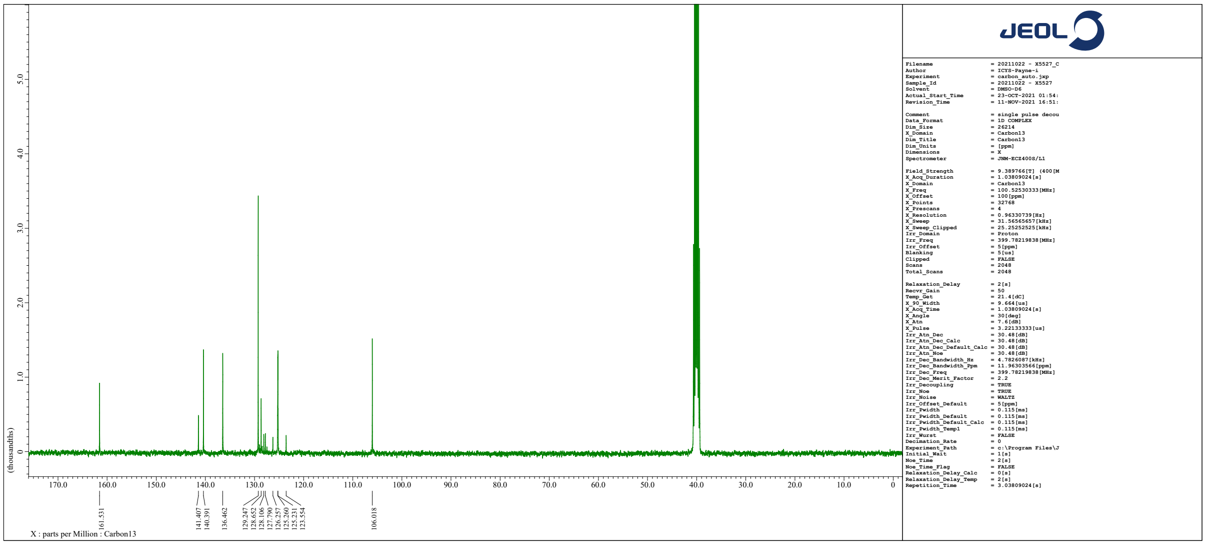


**Figure S35.** $^{13}$**C NMR spectrum of F in DMSO-*d*$_6$.**

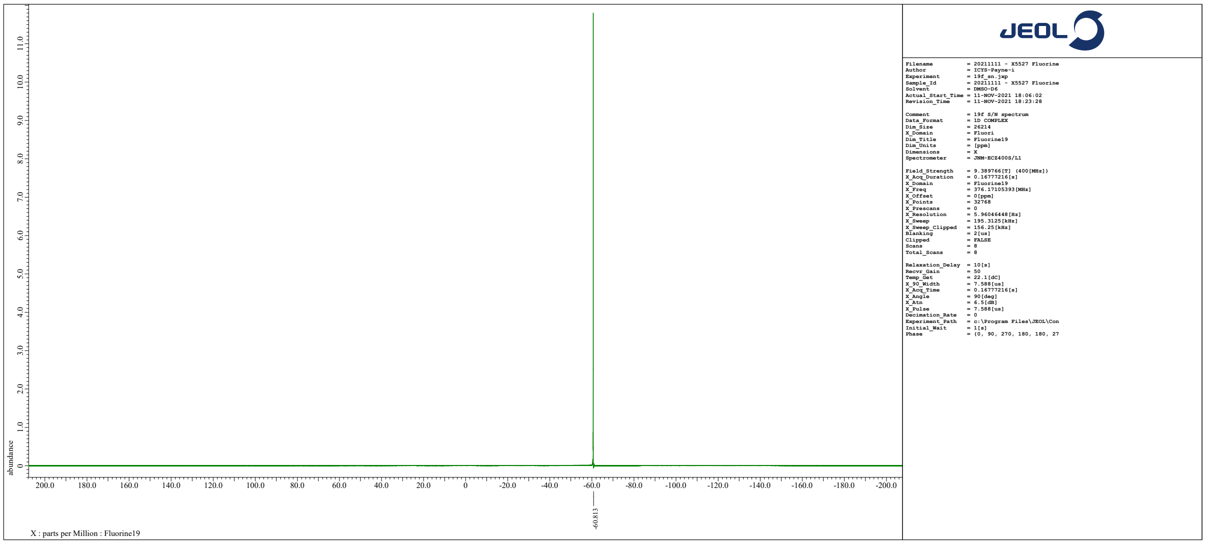


**Figure S36. $^{19}$F NMR spectrum of F in DMSO-$d_6$.**

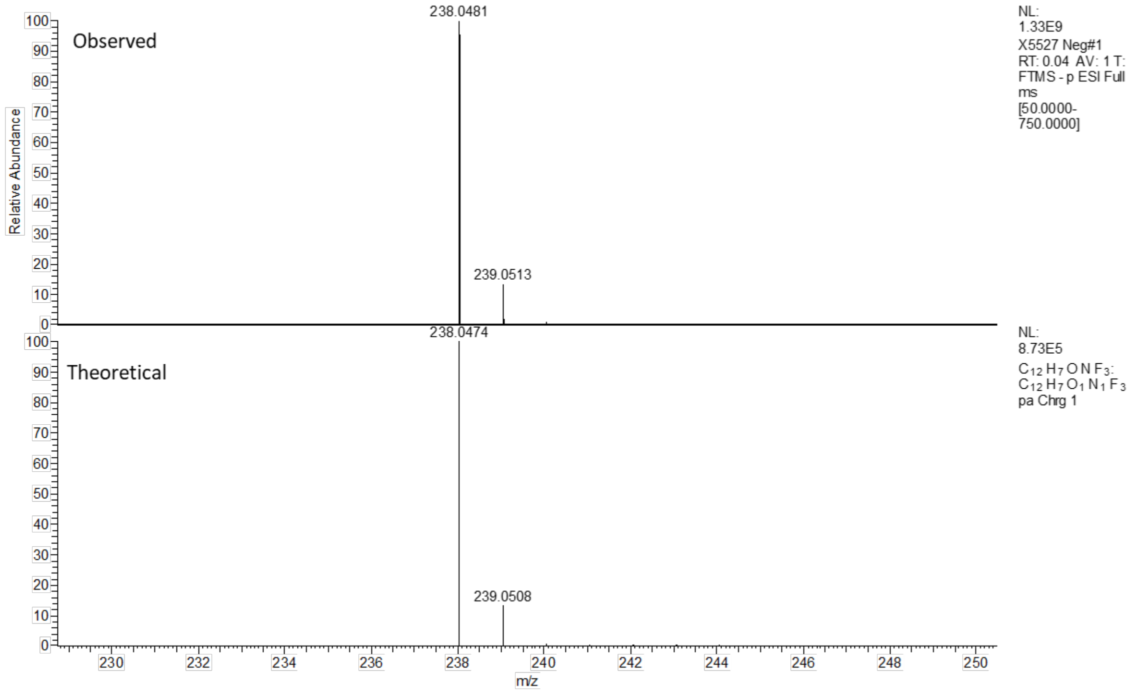


**Figure S37. HRMS analysis of compound F.**

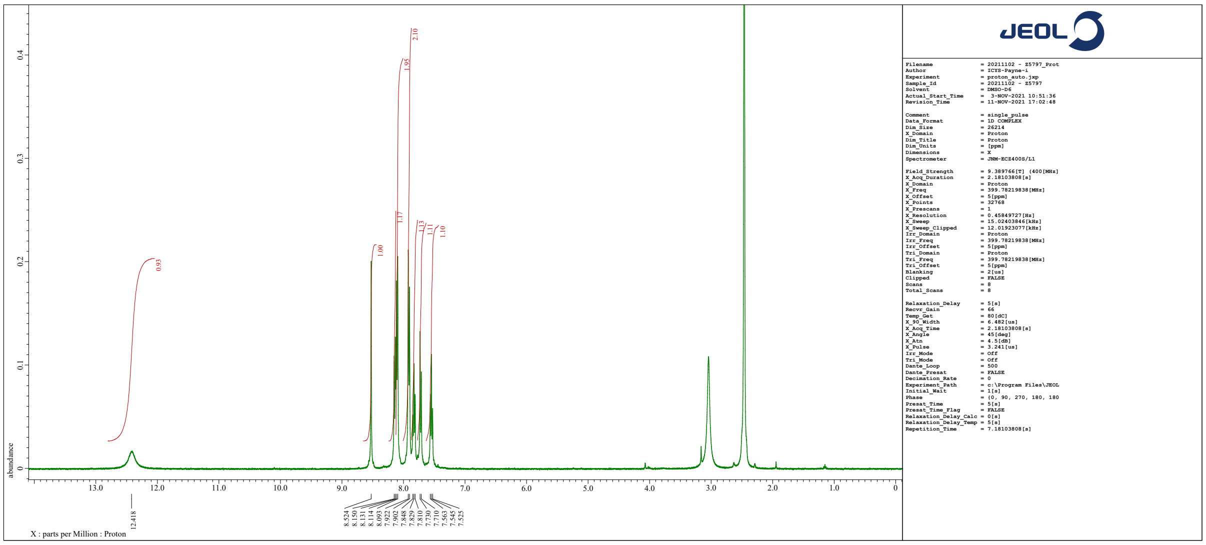


**Figure S38. ¹H NMR spectrum of G in at 80 °C DMSO-$d_6$.**

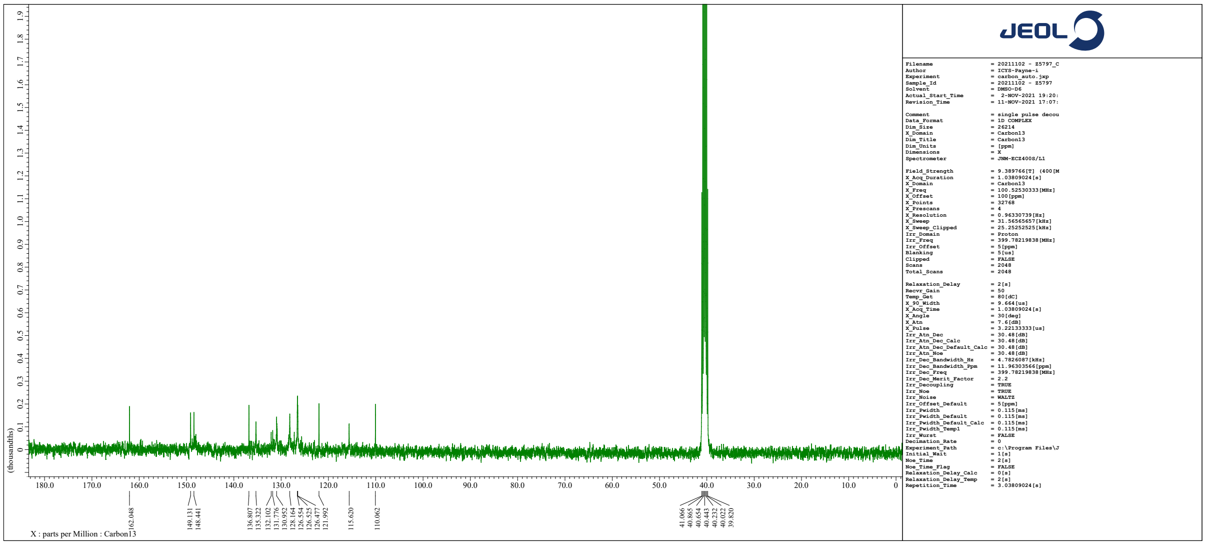


**Figure S39. ¹³C NMR spectrum of G at 80 °C in DMSO-$d_6$.**

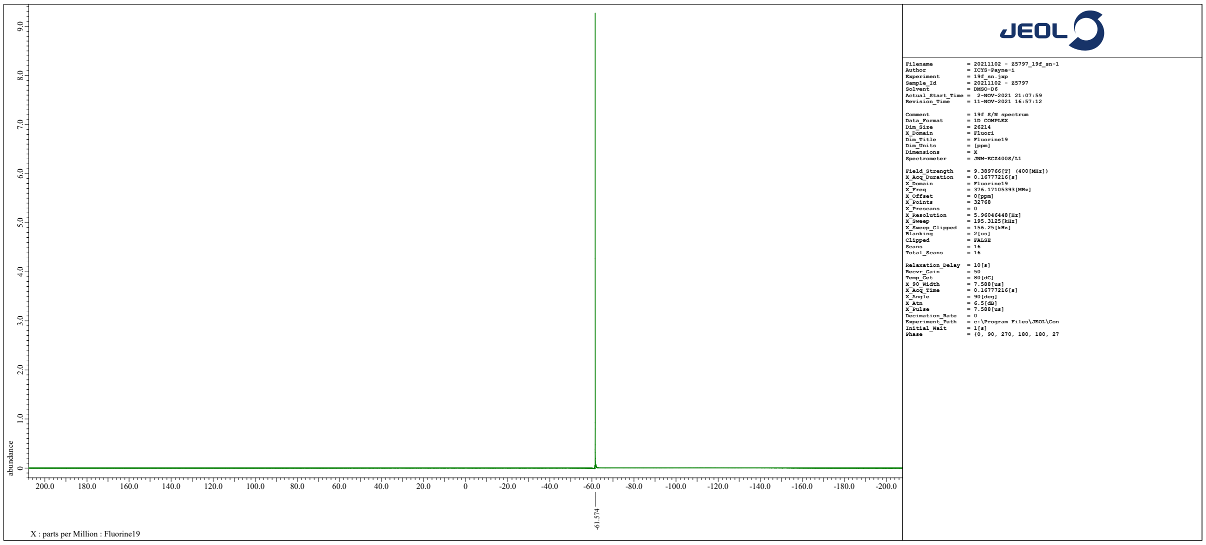


**Figure S40. $^{19}$F NMR spectrum of G at 80 °C in DMSO-$d_6$.**

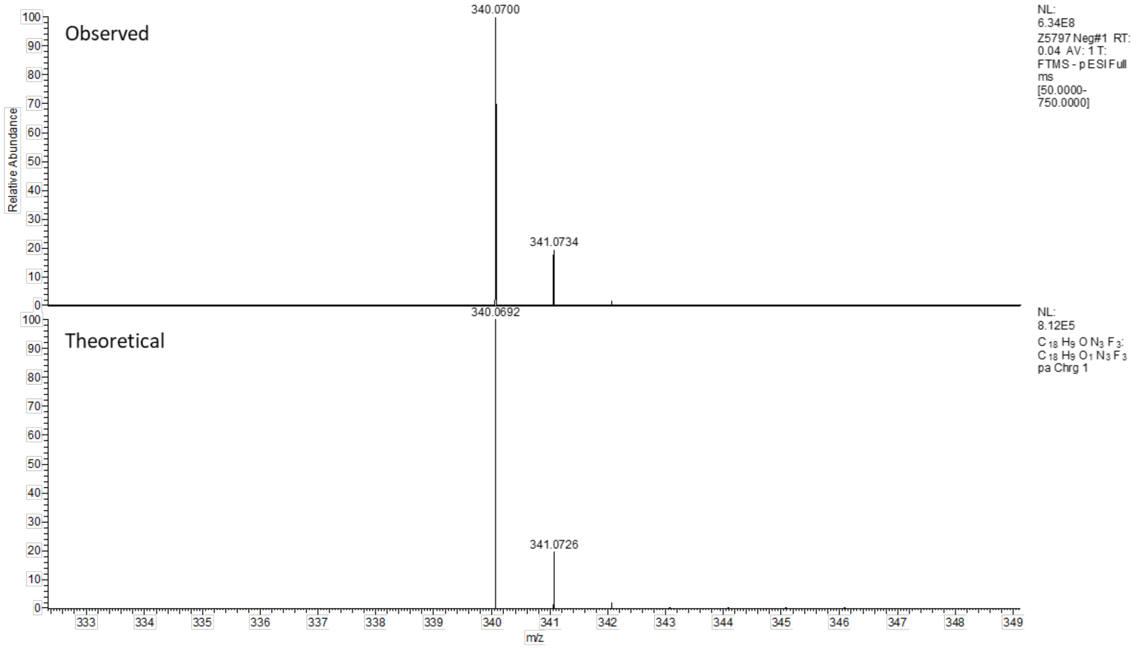


**Figure S41. HRMS analysis of compound G.**

## 6. Quantum yields of PC and A-G

Quantum yields ($\Phi$) in solid and solution states of **PC** and **A-G** molecules are summarized in Table S5. Absolute fluorescence quantum yields were determined with a Hamamatsu Photonics C-9920-02 calibrated integrating sphere system.

**Table S5.** Absolute fluorescence quantum yields of **PC** and **A-G**. Data are recoded in each solvent with a concentration of *10 μM or †1.0 μM.

| | **PC** | **A** | **B** | **C** | **D** | **E** | **F** | **G** |
|---|---|---|---|---|---|---|---|---|
| In DCM | 0.007* | — | 0.0† | 0.0† | 0.015† | 0.0† | 0.48† | — |
| In DMSO | 0.008* | — | — | — | — | — | — | — |
| In THF | — | 0.0† | — | — | — | — | — | 0.0† |
| Solid | 0.011 | 0.0 | 0.0 | 0.033 | 0.083 | 0.073 | 0.008 | 0.150 |

## 7. Cartesian Coordinates

Cartesian coordinates of optimized structures at B3LYP/6-31G* are tabulated, after their conformation search.

### 7.1. 3-phenyl coumarin

| | | | |
|---|---|---|---|
| C | 2.091476 | 0.284458 | 0.110934 |
| C | 2.753700 | -0.823651 | -0.327810 |
| C | 4.182141 | -0.951948 | -0.251821 |
| C | 4.901091 | 0.125666 | 0.293544 |
| C | 2.873193 | 1.413760 | 0.656942 |
| C | 4.899551 | -2.081890 | -0.690941 |
| C | 6.281895 | -2.125866 | -0.580001 |
| C | 6.975279 | -1.036800 | -0.028308 |
| C | 6.291383 | 0.092742 | 0.409767 |
| O | 4.254244 | 1.246239 | 0.723171 |
| O | 2.434681 | 2.467811 | 1.054478 |
| H | 2.191526 | -1.643302 | -0.768545 |
| H | 4.352033 | -2.919119 | -1.116461 |
| H | 6.827498 | -3.001082 | -0.919421 |
| H | 8.057464 | -1.072286 | 0.058077 |
| H | 6.806260 | 0.946750 | 0.837070 |
| C | 0.612981 | 0.383651 | 0.062324 |
| C | -0.043337 | 1.591207 | -0.236066 |
| C | -0.169837 | -0.763258 | 0.287196 |
| C | -1.433237 | 1.636868 | -0.328056 |
| H | 0.538498 | 2.490865 | -0.391493 |
| C | -1.559089 | -0.713378 | 0.197085 |
| H | 0.314301 | -1.696230 | 0.562978 |
| C | -2.197303 | 0.488199 | -0.115019 |
| H | -1.920984 | 2.578292 | -0.566425 |
| H | -2.142842 | -1.610915 | 0.383548 |
| H | -3.281129 | 0.530889 | -0.181619 |

### 7.2. PC

| | | | |
|---|---|---|---|
| O | 1.725470 | 1.594704 | -2.348906 |
| C | 2.444496 | 1.631991 | -1.375588 |
| O | 3.793407 | 1.854439 | -1.598256 |
| C | 4.714810 | 1.975603 | -0.598642 |
| C | 6.039766 | 2.214770 | -0.962959 |
| C | 6.994762 | 2.352811 | 0.039112 |
| C | 6.634419 | 2.255869 | 1.393074 |
| C | 5.313876 | 2.017421 | 1.743230 |
| C | 4.326781 | 1.869453 | 0.748145 |
| C | 2.941959 | 1.614065 | 1.021722 |
| C | 2.015911 | 1.467632 | 0.029056 |
| C | 0.604309 | 1.146313 | 0.311455 |
| C | 0.244017 | 0.420738 | 1.497195 |
| N | -0.984153 | 0.119851 | 1.852190 |
| C | -1.984228 | 0.518474 | 1.036597 |
| C | -3.380617 | 0.389717 | 1.081111 |
| C | -4.206447 | -0.249209 | 2.108639 |
| C | -3.652151 | -0.905045 | 3.221698 |
| C | -4.499426 | -1.492381 | 4.156068 |
| C | -5.878359 | -1.413879 | 3.961782 |
| N | -6.433818 | -0.793912 | 2.912384 |
| C | -5.612001 | -0.233610 | 2.023357 |
| C | -3.802977 | 1.026404 | -0.111462 |
| N | -2.810603 | 1.518091 | -0.858028 |
| N | -1.697709 | 1.207118 | -0.154270 |
| C | -0.432649 | 1.518491 | -0.522555 |
| H | 6.294573 | 2.289115 | -2.014768 |
| H | 8.029624 | 2.539112 | -0.232622 |
| H | 7.389934 | 2.367355 | 2.164622 |
| H | 5.023835 | 1.939622 | 2.787879 |
| H | 2.637892 | 1.551337 | 2.063248 |
| H | 1.030532 | 0.056453 | 2.154700 |
| H | -2.574858 | -0.945855 | 3.340592 |
| H | -4.095855 | -2.005654 | 5.024381 |
| H | -6.568002 | -1.863942 | 4.673981 |
| H | -6.101936 | 0.263449 | 1.187625 |
| H | -4.816336 | 1.148580 | -0.470345 |
| H | -0.318347 | 2.039606 | -1.461942 |

**7.3 PC1**

| | | | |
|---|---|---|---|
| O | 2.024084 | 2.441257 | 1.734665 |
| C | 2.661729 | 1.663064 | 1.061367 |
| O | 4.022978 | 1.896360 | 0.950590 |
| C | 4.877452 | 1.077661 | 0.270631 |
| C | 6.228830 | 1.422529 | 0.251155 |
| C | 7.120580 | 0.597262 | -0.426347 |
| C | 6.671839 | -0.563214 | -1.077651 |
| C | 5.325607 | -0.895700 | -1.051785 |
| C | 4.399990 | -0.075205 | -0.376284 |
| C | 2.991878 | -0.340809 | -0.306051 |
| C | 2.124286 | 0.483867 | 0.350908 |
| C | 0.669784 | 0.240775 | 0.370237 |
| C | -0.148423 | 0.656029 | 1.404805 |
| N | -1.464351 | 0.350027 | 1.346475 |
| N | -2.383078 | 0.674059 | 2.290521 |
| C | -3.519684 | 0.163224 | 1.812613 |
| C | -3.382381 | -0.495553 | 0.568331 |
| C | -2.020260 | -0.366425 | 0.271032 |
| N | -1.245789 | -0.756890 | -0.764107 |
| C | 0.033000 | -0.460344 | -0.708241 |
| C | -4.452850 | -1.144194 | -0.190270 |
| N | -5.677460 | -1.103266 | 0.376046 |
| C | -6.689970 | -1.679276 | -0.275115 |
| C | -6.559296 | -2.321956 | -1.507282 |
| C | -5.292148 | -2.362261 | -2.089612 |
| C | -4.221413 | -1.768868 | -1.429062 |
| H | 6.553122 | 2.321785 | 0.763963 |
| H | 8.175169 | 0.855887 | -0.448315 |
| H | 7.379009 | -1.199136 | -1.601199 |
| H | 4.967123 | -1.791030 | -1.553166 |
| H | 2.624860 | -1.242667 | -0.788876 |
| H | 0.178291 | 1.218462 | 2.267415 |
| H | -4.435694 | 0.276173 | 2.375175 |
| H | 0.626489 | -0.755770 | -1.571003 |
| H | -7.661094 | -1.624074 | 0.215549 |
| H | -7.421629 | -2.772708 | -1.989099 |
| H | -5.138306 | -2.850533 | -3.048598 |
| H | -3.221274 | -1.777840 | -1.848015 |

**7.4 PC2**

| | | | |
|---|---|---|---|
| O | 0.255918 | -1.562909 | -0.926171 |
| C | 1.315622 | -1.051598 | -0.640111 |
| O | 2.454447 | -1.792312 | -0.944184 |
| C | 3.723994 | -1.373710 | -0.687392 |
| C | 3.956030 | -0.125579 | -0.082209 |
| C | 5.288266 | 0.263017 | 0.169304 |
| C | 6.339373 | -0.571275 | -0.177653 |
| C | 6.078738 | -1.812617 | -0.782756 |
| C | 4.774623 | -2.221584 | -1.040646 |
| C | 2.813860 | 0.672064 | 0.241630 |
| C | 1.535095 | 0.264336 | -0.013469 |
| C | 0.390674 | 1.145383 | 0.337359 |
| N | 0.701865 | 2.399560 | 0.657580 |
| C | -0.279602 | 3.240397 | 1.019094 |
| C | -0.310076 | 4.592486 | 1.408509 |
| C | -1.675385 | 4.840920 | 1.662453 |
| N | -2.472228 | 3.785282 | 1.461734 |
| N | -1.619141 | 2.812548 | 1.070172 |
| C | -1.953396 | 1.540955 | 0.739975 |
| C | -0.961848 | 0.677547 | 0.362551 |
| C | 0.777212 | 5.562529 | 1.535703 |
| N | 0.426054 | 6.775850 | 2.013521 |
| C | 1.378760 | 7.700617 | 2.145178 |
| C | 2.720042 | 7.491454 | 1.819821 |
| C | 3.080038 | 6.238516 | 1.323245 |
| C | 2.103040 | 5.259618 | 1.176209 |
| H | 5.474620 | 1.225696 | 0.637894 |
| H | 7.363313 | -0.267813 | 0.017186 |
| H | 6.904422 | -2.464321 | -1.053517 |
| H | 4.552371 | -3.176040 | -1.505772 |
| H | 2.960239 | 1.640501 | 0.709910 |
| H | -2.101694 | 5.778728 | 1.989362 |
| H | -3.006635 | 1.294462 | 0.801582 |
| H | -1.195154 | -0.337924 | 0.081900 |
| H | 1.052019 | 8.664765 | 2.533316 |
| H | 3.450632 | 8.283998 | 1.949953 |
| H | 4.110899 | 6.026679 | 1.050252 |
| H | 2.339989 | 4.276172 | 0.786885 |

**7.5. PC3**

| | | | |
|---|---|---|---|
| O | -2.981290 | 0.731241 | 2.882298 |
| C | -2.922864 | 0.475805 | 1.699466 |
| O | -4.074285 | -0.064326 | 1.134395 |
| C | -4.186381 | -0.411752 | -0.177272 |
| C | -3.103632 | -0.235956 | -1.057433 |
| C | -3.264391 | -0.611219 | -2.407730 |
| C | -4.467262 | -1.140379 | -2.848247 |
| C | -5.533019 | -1.305341 | -1.946965 |
| C | -5.401420 | -0.944018 | -0.610202 |
| C | -1.900325 | 0.317043 | -0.519551 |
| C | -1.776886 | 0.672987 | 0.794755 |
| C | -0.498297 | 1.250590 | 1.285666 |
| N | 0.420516 | 1.489968 | 0.354003 |
| C | 1.607063 | 1.992061 | 0.722812 |
| C | 2.772584 | 2.341061 | 0.011971 |
| C | 3.650141 | 2.790889 | 1.020932 |
| N | 3.138980 | 2.751337 | 2.258652 |
| N | 1.895721 | 2.261447 | 2.070805 |
| C | 0.968189 | 2.031140 | 3.035431 |
| C | -0.247865 | 1.524680 | 2.669779 |
| C | 3.025570 | 2.254934 | -1.427297 |
| C | 4.331244 | 2.214452 | -1.943319 |
| C | 4.524415 | 2.147346 | -3.319105 |
| C | 3.405056 | 2.109179 | -4.151227 |
| N | 2.148542 | 2.143033 | -3.689478 |
| C | 1.976260 | 2.219602 | -2.367030 |
| H | -2.431651 | -0.476315 | -3.092703 |
| H | -4.587831 | -1.427164 | -3.888264 |
| H | -6.474297 | -1.720356 | -2.295687 |
| H | -6.212059 | -1.063491 | 0.100638 |
| H | -1.046003 | 0.463099 | -1.173488 |
| H | 4.656261 | 3.169950 | 0.894399 |
| H | 1.266636 | 2.269631 | 4.049247 |
| H | -1.010123 | 1.335172 | 3.409884 |
| H | 5.185508 | 2.219684 | -1.271516 |
| H | 5.525102 | 2.114122 | -3.740382 |
| H | 3.519259 | 2.047975 | -5.232296 |
| H | 0.947835 | 2.256786 | -2.018744 |

**7.6. V**

| | | | |
|---|---|---|---|
| O | 3.375093 | 2.706237 | -1.681708 |
| C | 3.548773 | 1.686117 | -1.055705 |
| O | 4.863718 | 1.359906 | -0.755568 |
| C | 5.232968 | 0.247235 | -0.058524 |
| C | 4.261724 | -0.656430 | 0.406303 |
| C | 4.694525 | -1.791957 | 1.121926 |
| C | 6.044870 | -2.003488 | 1.356323 |
| C | 6.993291 | -1.083558 | 0.879961 |
| C | 6.593625 | 0.045026 | 0.171086 |
| C | 2.890702 | -0.361458 | 0.120278 |
| C | 2.502661 | 0.751266 | -0.577808 |
| C | 1.109226 | 1.059134 | -0.872664 |
| C | 0.565725 | 2.127632 | -1.560458 |
| C | -0.845843 | 2.090005 | -1.648869 |
| C | -1.419681 | 0.996272 | -1.033814 |
| S | -0.175494 | -0.016485 | -0.323246 |
| C | -2.816993 | 0.642700 | -0.930931 |
| C | -3.383227 | -0.452138 | -0.331780 |
| C | -4.813283 | -0.489711 | -0.416769 |
| C | -5.709898 | -1.454848 | 0.081290 |
| C | -7.073445 | -1.290802 | -0.117250 |
| C | -7.570202 | -0.171832 | -0.810222 |
| C | -6.706735 | 0.797056 | -1.313134 |
| C | -5.334461 | 0.630643 | -1.112479 |
| S | -4.048827 | 1.702897 | -1.643717 |
| H | 3.952396 | -2.497676 | 1.486229 |
| H | 6.369696 | -2.880300 | 1.908185 |
| H | 8.050289 | -1.251447 | 1.064835 |
| H | 7.307238 | 0.769550 | -0.206776 |
| H | 2.142346 | -1.063112 | 0.481290 |
| H | 1.180837 | 2.908293 | -1.983658 |
| H | -1.427754 | 2.852299 | -2.156084 |
| H | -2.799991 | -1.222756 | 0.162073 |
| H | -5.328800 | -2.320351 | 0.616966 |
| H | -7.766928 | -2.033983 | 0.266098 |
| H | -8.640963 | -0.060564 | -0.955840 |
| H | -7.092179 | 1.660226 | -1.847939 |
